# Supplementary figures and images for: Mechanistic insights into global suppressors of protein folding defects
Source: PLoS Genet. 2022 Aug 29;18(8):e1010334. doi: 10.1371/journal.pgen.1010334 (PMC9491731; doi:10.1371/journal.pgen.1010334)

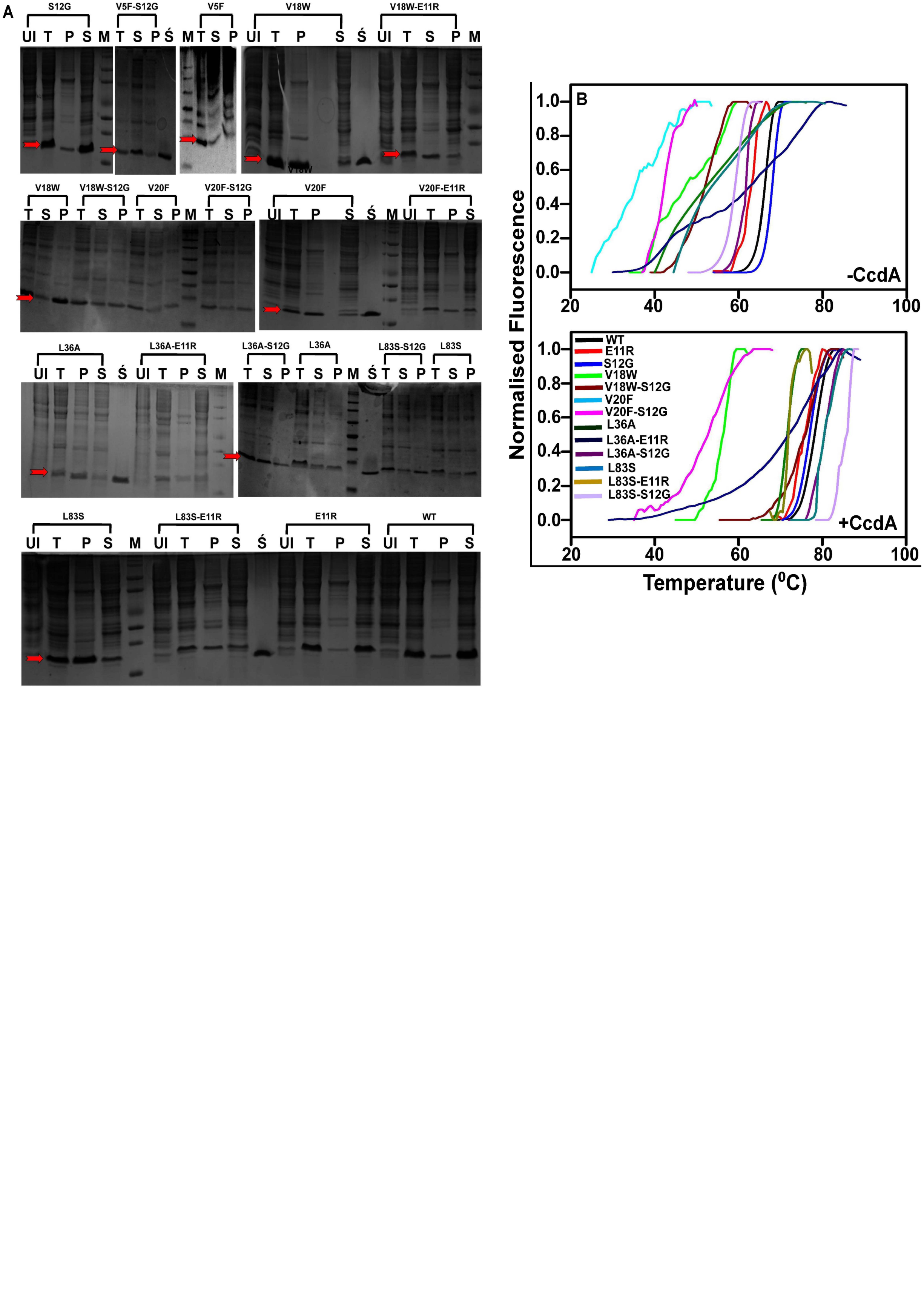

Supplement: S1 Fig — (A-B) Solubility and thermal stabilities of CcdB mutants, in presence and absence of the suppressors. (A) In vivo solubility estimates for CcdB mutants. UI, T, S and P are uninduced, total cell lysate, supernatant and pellet respectively. Ś is the purified CcdB WT protein used as standard and M is the molecular weight marker lane. The relative estimates of protein present in the soluble fraction and inclusion bodies for all mutants are shown in S1 Table. The red arrow indicates the band for the induced protein. (B) Thermal unfolding profiles of purified WT CcdB and CcdB mutants in the absence and presence of 8 μM CcdA peptide (45–72) measured by a thermal shift assay (TSA). L83S-E11R and V20F are omitted in the top and bottom panels as they do not show clear thermal transitions in the absence and presence of CcdA respectively. (TIFF) [file pgen.1010334.s001.tiff]

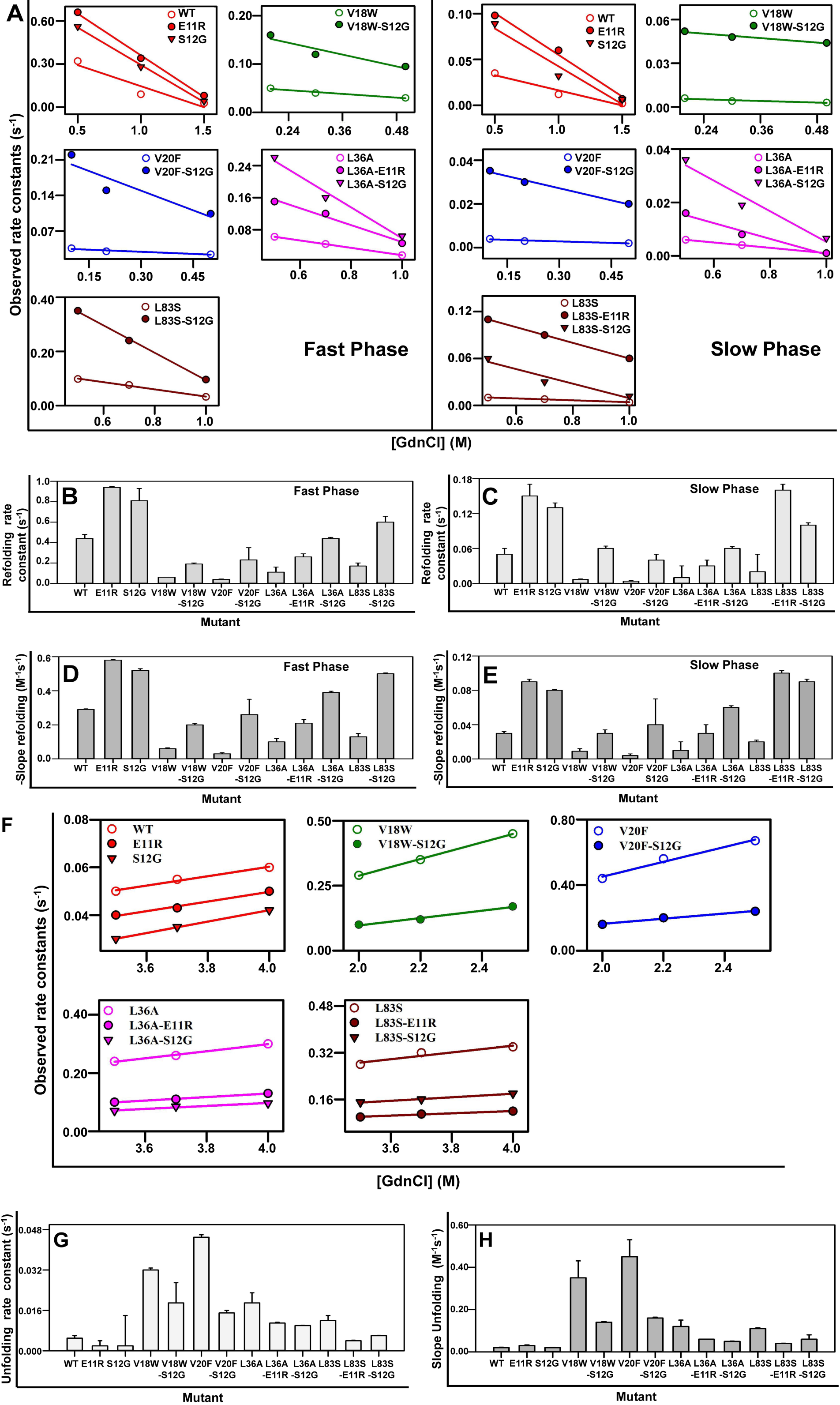

Supplement: S2 Fig — (A-E) Representative refolding rate constants of fast phase (left side) and slow phase (right panel) and (F-H) unfolding rate constants at 5 μM protein concentration of the WT and mutants are shown. The experimental rate constants (for refolding and unfolding) obtained at increasing final GdnCl concentrations are shown in dots, while fits are shown in solid lines. For refolding kinetics, the extrapolated rate constants and the magnitude of refolding m-values of the transition states at zero denaturant concentration of fast phase (B and D respectively) and slow phase (C and E respectively) are shown. Suppressor mutations significantly accelerate the refolding rate constants. For unfolding kinetics, the extrapolated rate constants (G) and the magnitude of unfolding m-values (H) at zero denaturant concentration are shown. Suppressor mutations decrease the unfolding rate constants. The error bars wherever shown represent the standard deviation from two independent experiments, each performed in duplicates (see also S4 Table). (TIFF) [file pgen.1010334.s002.tiff]

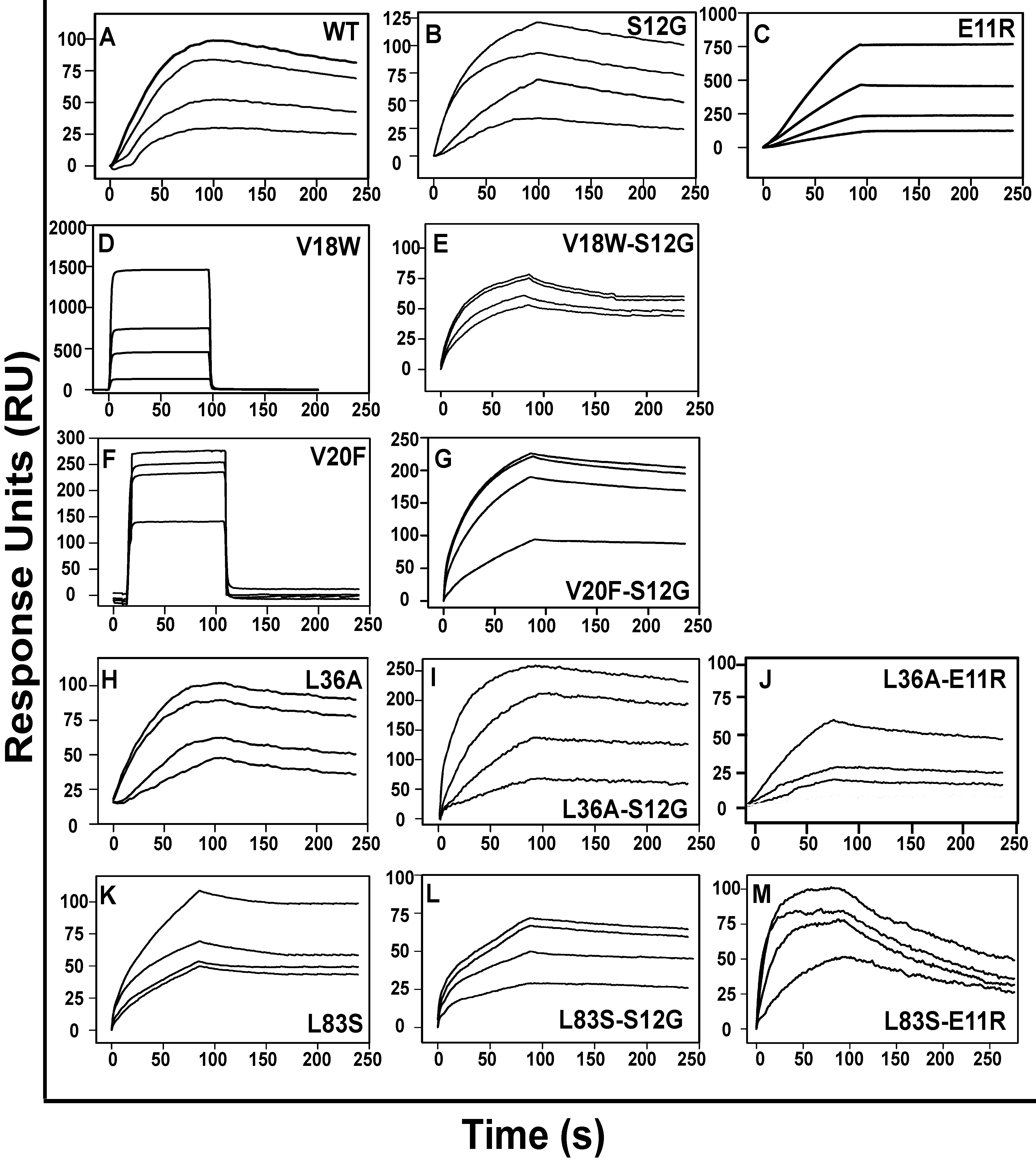

Supplement: S4 Fig — Overlays show the binding kinetics with analyte concentration increasing from the bottom to the top curve in all cases of (A) WT CcdB (3, 6, 12.5, 25 nM); (B) S12G (3, 6, 25, 50 nM); (C) E11R (50, 100, 200, 500 nM); (D) V18W (200, 500, 1000, 2000 nM); (E) V18W-S12G (200, 500, 1000, 2000 nM); (F) V20F (200, 1000, 2000, 5000 nM); (G) V20F-S12G (100, 200, 500, 1000 nM); (H) L36A (12.5, 25, 100, 200 nM); (I) L36A-S12G (3, 6, 12.5, 25 nM); (J) L36A-E11R (10, 21.4, 32, 64.2 nM); (K) L83S (200, 500, 1000, 2000 nM); (L) L83S-S12G (200, 500, 1000, 2000 nM); (M) L83S-E11R (50, 100, 200, 500 nM). The ligand GyrA14 was immobilized on the CM5 chip by standard amine coupling. Binding was measured by passing varying concentrations of the analyte (CcdB proteins) over the ligand (GyrA14) immobilised chip and the data was fitted to the 1:1 Langmuir Interaction model to obtain the kinetic parameters (S1 Table). (TIFF) [file pgen.1010334.s004.tiff]

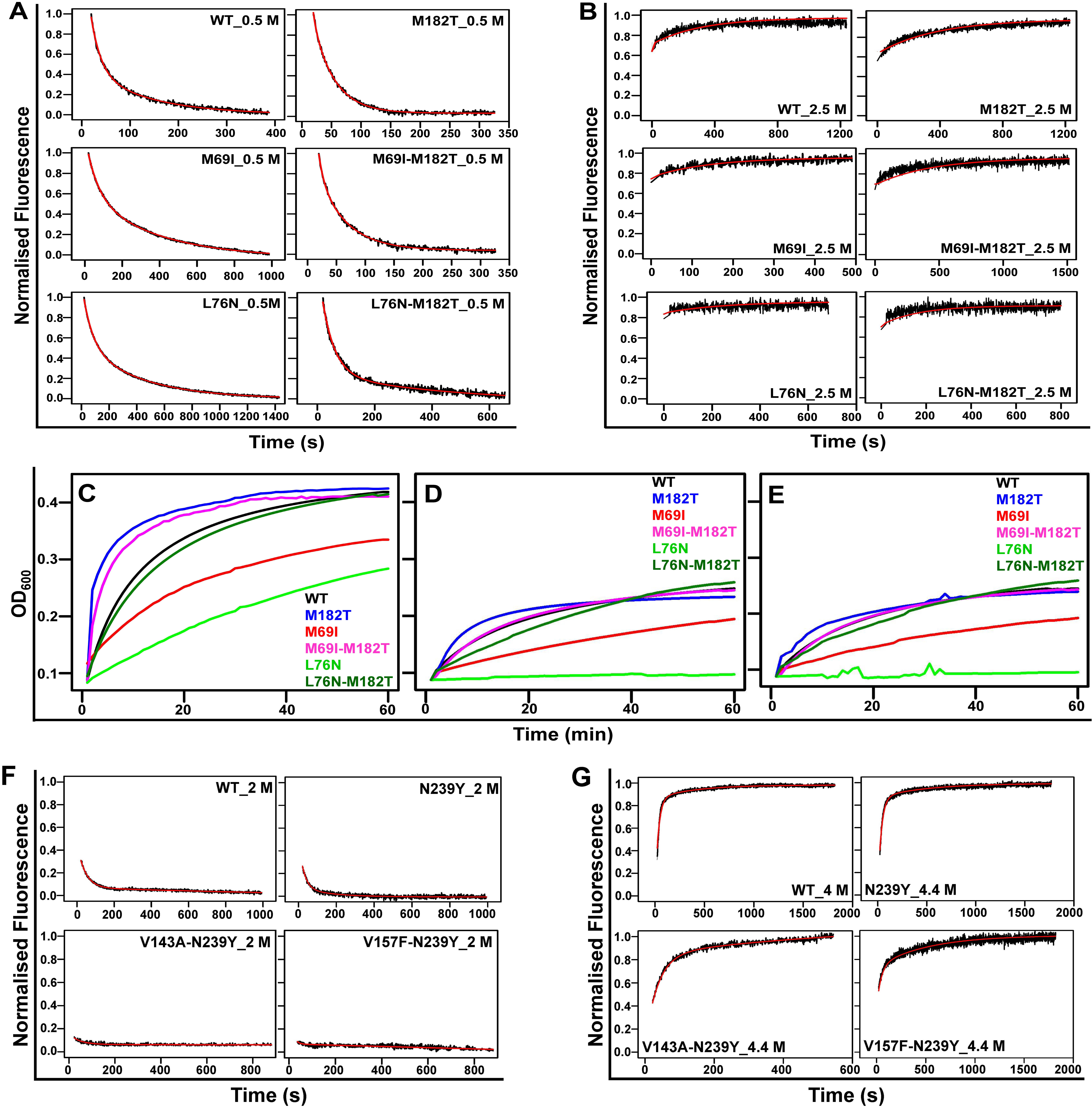

Supplement: S5 Fig — (A) TEM-1 proteins exhibit biphasic refolding kinetics with a fast and slow phase whereas (B) unfolding follows single exponential kinetics. Representative kinetic traces at 5 μM protein concentrations of WT and TEM-1 mutants are shown in presence of 0.5 M GdnCl for refolding and 2.5 M GdnCl for unfolding. The experimental kinetic traces obtained at the indicated GdnCl concentrations are shown in black, while the fits are shown in red. The measured kinetic parameters are listed in S6 Table. The lactamase activities of the WT and mutants in the following conditions–(C) native, (D) native proteins in 0.5 M GdnCl and (E) refolded proteins in 0.5 M GdnCl, were assayed by observing the rate of nitrocefin hydrolysis (50 μM) at 486 nm at 25°C at a protein concentration of 10 nM. The M182T suppressor rescues the activity of M69I and L76N mutants. (F) p53-DBD proteins exhibit biphasic refolding kinetics with significant burst, fast and slow phases whereas (G) unfolding of p53-DBD follows biphasic exponential kinetics with burst, fast and slow phases. Representative kinetic traces at 5 μM protein concentration of WT and p53-DBD mutants are shown in the presence of 2 M Urea for refolding and 4.4 M Urea for unfolding. The experimental kinetic traces obtained are shown in black, while the fits are shown in red. The measured kinetic parameters are listed in S7 Table. (TIFF) [file pgen.1010334.s005.tiff]

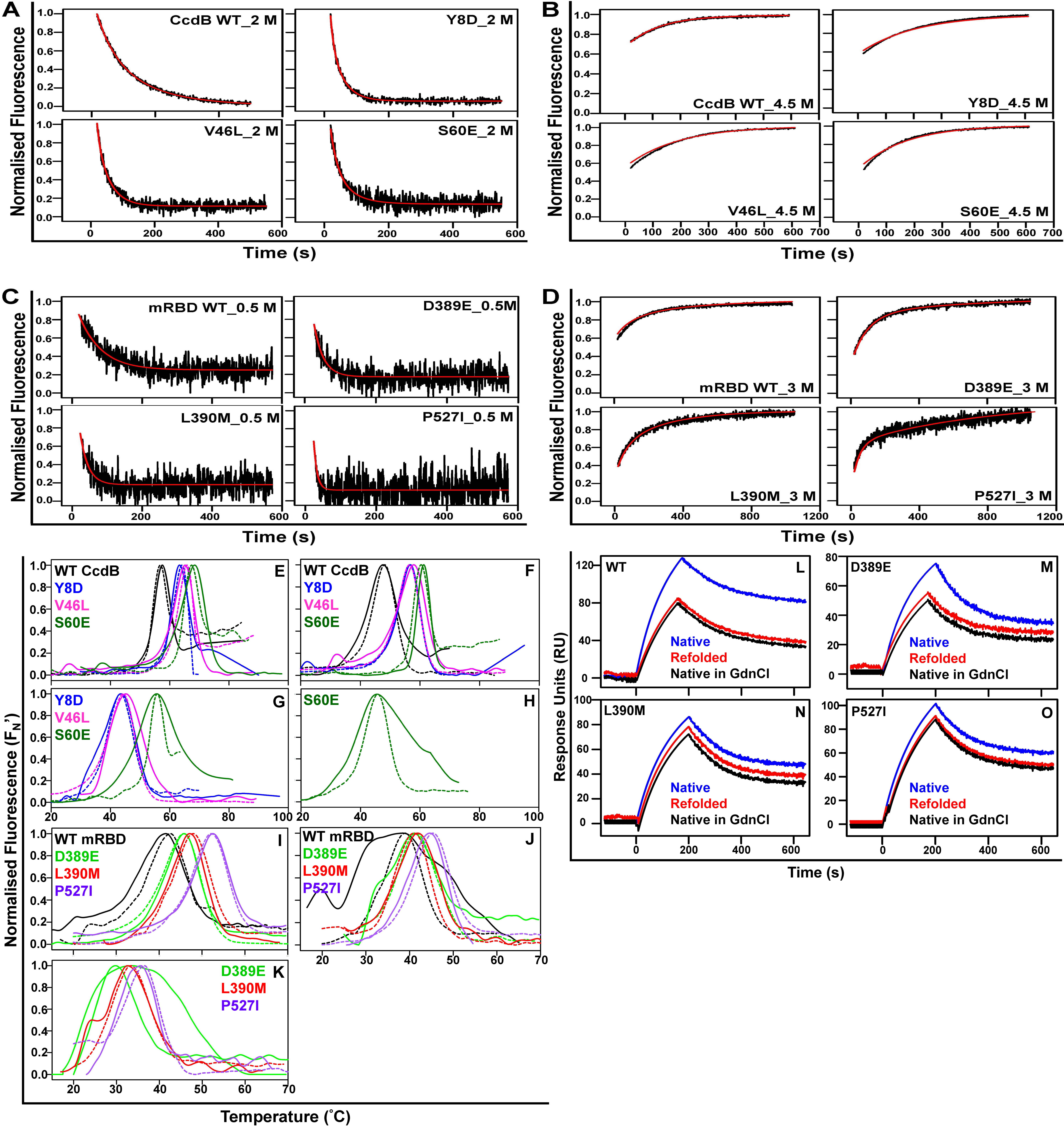

Supplement: S6 Fig — (A) Biphasic refolding kinetics with a fast and slow phase and (B) single exponential unfolding kinetics of CcdB mutant proteins. Representative kinetic traces at 5 μM protein concentration of WT CcdB; Y8D; V46L; S60E are shown. (C) mRBD proteins exhibit single phase refolding kinetics with a significant burst phase whereas (D) unfolding of mRBD proteins follows biphasic exponential kinetics with burst, fast and slow phases. Representative kinetic traces at 5 μM protein concentration of WT mRBD; D389E; L390M; P527I are shown. The experimental kinetic traces obtained at different GdnCl concentrations are shown in black, while the fits are shown in red. The measured kinetic parameters are listed in S9 Table for CcdB and mRBD mutants. (E-H) Thermal denaturation traces of 5 μM of native CcdB proteins in (E) 1 M, (F) 2 M, (G) 3 M and (H) 4 M GdnCl (represented by dashed lines) and refolded CcdB proteins in the same concentrations of GdnCl (represented by solid lines). WT CcdB failed to show any transition at 3 M, whereas Y8D and V46L failed to show a thermal unfolding transition at 4 M. S60E showed a thermal transition at 4 M. (I-K) Thermal denaturation traces of 5 μM of native mRBD proteins in (I) 0.5 M, (J) 1 M and (K) 2 M (represented by dashed lines) and refolded mRBD proteins in same concentrations of GdnCl (represented by solid lines). mRBD WT failed to show any transition at 2 M, whereas the stabilised mutants show thermal unfolding upto 2M GdnCl. (L-O) Overlays show the ACE2-hFc binding of the WT and mRBD mutants in the following conditions–native, native in 0.5 M GdnCl and refolded in 0.5 M GdnCl for (L) WT mRBD, (M) D389E, (N) L390M and (O) P527I. The ligand ACE2-hFc was immobilized on Protein-G immobilised GLM sensor chip. Binding was measured by passing 50 nM of the analyte (mRBD proteins) over the ligand (ACE2-hFc) immobilised chip. (TIFF) [file pgen.1010334.s006.tiff]

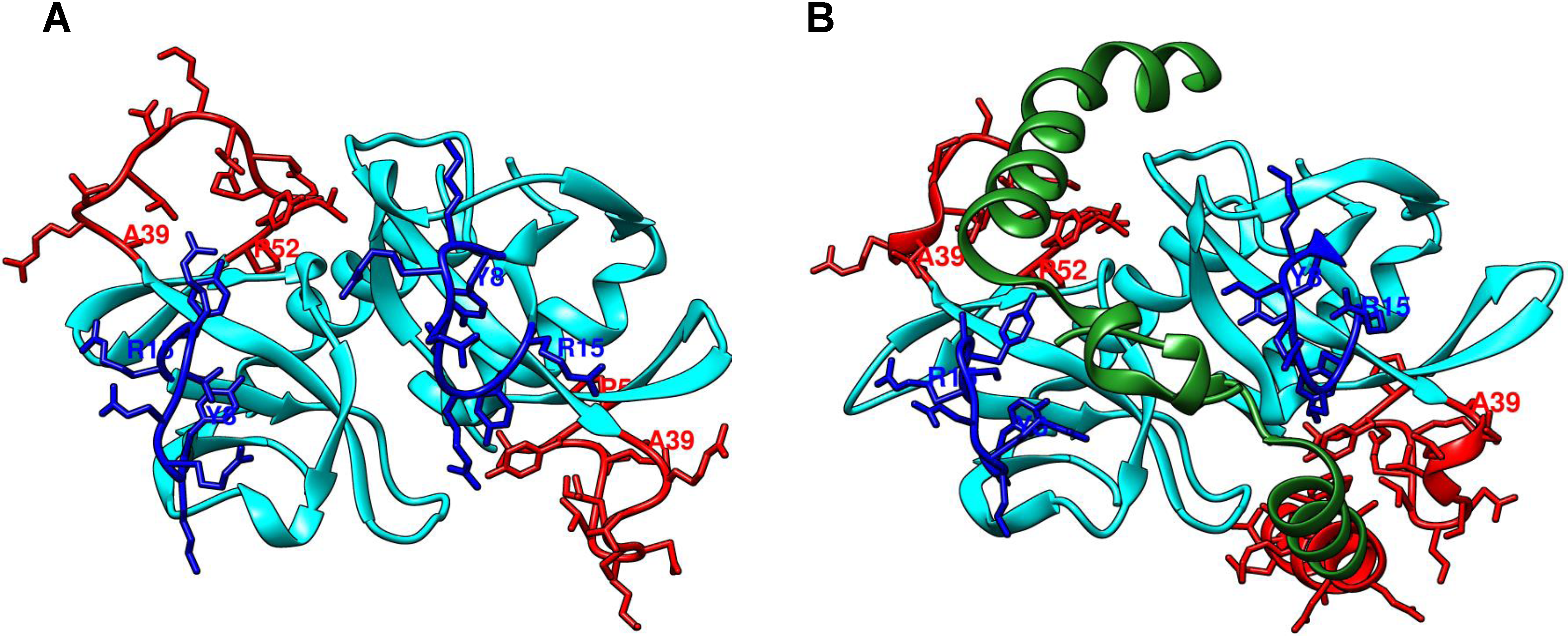

Supplement: S7 Fig — The crystal structures of CcdB in (A) free (PDB ID:3VUB) and (B) CcdA bound state (PDB ID:3G7Z) are shown (2,3). The CcdB dimer is shown in cyan and the 8–15 loop and 39–52 loop are shown in blue and red respectively. The CcdA dimer is shown in green in the CcdA bound state. The conformation of the residues 8–15 and 39–52 change in the CcdA bound state as most of these residues of these loops are involved in direct interaction with CcdA. (TIFF) [file pgen.1010334.s007.tiff]

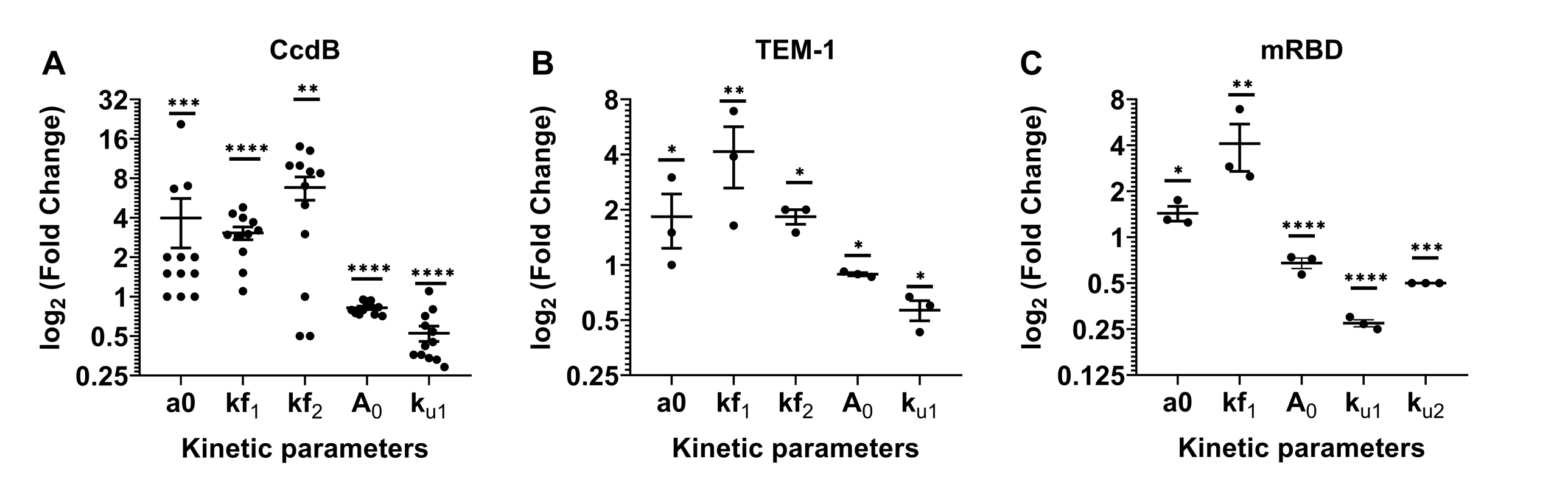

Supplement: S8 Fig — (A-C) Log2 fold change of various kinetic parameters (Mean±SEM) for the suppressor mutations in the background of WT or inactive mutant for (A) CcdB, (B) TEM-1 β-lactamase and (C) mRBD proteins. Mann Whitney non-parametric test was performed for each of these parameters. The mean of the distributions of the values for each of the parameters are significantly higher than log2(2) for refolding. P value indicated with *, ** and *** indicates < 0.05, < 0.005 and < 0.0005 respectively. a0, kf1, kf2, A0, ku1, ku2 are the burst phase amplitude for refolding, rate constant of fast phase of refolding, rate constant of slow phase of refolding, amplitude of burst phase of unfolding, rate constant of fast phase of unfolding, and the rate constant of slow phase of unfolding respectively. (TIF) [file pgen.1010334.s008.tif]

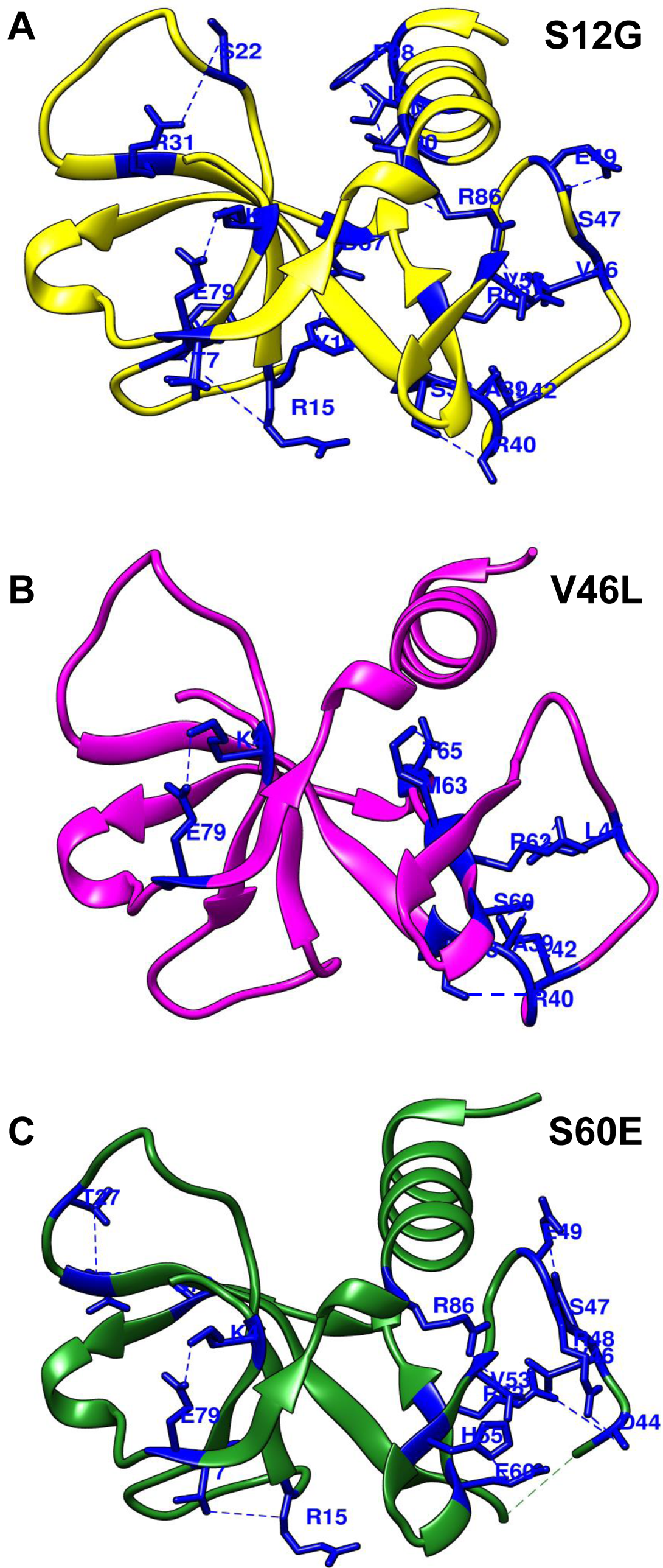

Supplement: S9 Fig — Detailed intramolecular interactions between residues in three crystal structures of CcdB suppressor mutants, namely, (A) S12G, (B) V46L and (C) S60E (Related to Fig 6). The CcdB chains are coloured in yellow in S12G, magenta in V46L and green in S60E, while the interacting residues are coloured in blue. Additional hydrogen-bonded interactions present in the suppressor, but absent in the WT (blue dotted lines) between polar and charged residues are shown. (TIFF) [file pgen.1010334.s009.tiff]
