# Supplementary material for: Mechanistic insights into global suppressors of protein folding defects
Source: PLoS Genet. 2022 Aug 29;18(8):e1010334. doi: 10.1371/journal.pgen.1010334 (PMC9491731; doi:10.1371/journal.pgen.1010334)
Supplement: S1 Table — Binding affinities of refolded proteins and proteins in GdnCl were also measured by MST. SPR experiments were carried out at 25°C, pH 7.4 and MST experiments were carried out at 25°C, pH 8.4. 1Reported standard errors are derived from two independent experiments, each performed in duplicates. 2Mutants could not be purified. 3,4 Refolded in 0.1 M GdnCl. All the other proteins were refolded in 1.5 M GdnCl. NB: No Binding. (DOCX) [file pgen.1010334.s010.docx]

**S1_Table.** **Fractional solubilities, thermal stabilities (determined by nanoDSF) and GyrA14 binding affinities (determined by SPR and MST) of different CcdB mutants^1^ (Related to Figs 1 and 2).** Binding affinities of refolded proteins and proteins in GdnCl were also measured by MST. SPR experiments were carried out at 25 °C, pH 7.4 and MST experiments were carried out at 25 °C, pH 8.4.

| **Mutants** | **Fraction**  **Soluble** | **T_m_**  **(°C)** | **K_D_**  **(nM)**  **SPR** | **MST Studies** | | |
| --- | --- | --- | --- | --- | --- | --- |
|  |  |  |  | **K_D_ (nM)**  **Native** | **K_D_ (nM)**  **Native in GdnCl** | **K_D_ (nM)**  **Refolded** |
| **WT** | 1.0 | 66±0.8 | 1.4±0.6 | 2.2±0.6 | 41.8±3.2 | 32.2±2.7 |
| **E11R** | 0.9 | 64±1.0 | 2.5±0.3 | 1.2±0.3 | 3.2±0.3 | 2.4±0.4 |
| **S12G** | 0.9 | 68±0.8 | 2.6±1.4 | 2.3±0.8 | 74.5±10 | 99±15 |
| **V18W** | 0.2 | 54±09 | NB | 2046.9±132 | 2241.8^3^±168 | 2577.4^a^±196 |
| **V18W-E11R^2^** | 0.6 | - | - | - | - | - |
| **V18W-S12G** | 0.6 | 66±0.9 | 57±13.2 | 30.2±4 | 132.2±12 | 149.3±16 |
| **V20F** | 0.3 | 47±0.8 | NB | 623.5±92 | 1465.1^4^±117 | 1078.0^b^±102 |
| **V20F-E11R^2^** | 0.5 | - | - | - | - | - |
| **V20F-S12G** | 0.4 | 60±0.8 | 12.7±4 | 20.9±12 | 130.9±48 | 103.6±29 |
| **L36A** | 0.3 | 58±1.0 | 9.5±4 | 17.5±7 | 125.2±30 | 162.0±54 |
| **L36A-E11R** | 0.6 | 63±1.0 | 3.0±1.3 | 13±1.3 | 16.0 ±1.3 | 1.3±1.0 |
| **L36A-S12G** | 0.7 | 63±0.2 | 0.4±0.7 | 0.3±1.4 | 1.5±0.6 | 2.3±2 |
| **L83S** | 0.4 | 59±0.8 | 75.0±15.3 | 88.7±19 | 175.3±63 | 199.4±45 |
| **L83S-E11R** | 0.5 | 67±0.5 | 6.8±2.9 | 0.30±0.1 | 16±2.9 | 0.4±0.1 |
| **L83S-S12G** | 0.5 | 66±0.7 | 7.2±11.3 | 4.12±2.3 | 11.9±9.4 | 13±7.2 |

^1^Reported standard errors are derived from two independent experiments, each performed in duplicates.

^2^Mutants could not be purified.

^3,4^ Refolded in 0.1 M GdnCl. All the other proteins were refolded in 1.5 M GdnCl.

NB: No Binding.
