# Supplementary material for: Mechanistic insights into global suppressors of protein folding defects
Source: PLoS Genet. 2022 Aug 29;18(8):e1010334. doi: 10.1371/journal.pgen.1010334 (PMC9491731; doi:10.1371/journal.pgen.1010334)
Supplement: S2 Table — Residual activity after high temperature incubation of CcdB mutants was determined by SPR at room temperature. 1Reported standard errors are derived from two independent experiments, each performed in duplicates. 2,3 Could not be refolded in 1.5 M GdnCl. All the other proteins were refolded in 1.5 M GdnCl.-Not determined. (DOCX) [file pgen.1010334.s011.docx]

**S2_Table.** **Thermodynamic stability parameters (C_m_, ΔG⁰, m_equi_), following chemical denaturation and melting temperatures of refolded and native proteins in presence of 1.5 M GdnCl (T_mRefold_ , T_mGdnCl_) determined by nanoDSF, of different CcdB mutants^1^ (Related to Fig 2).** Residual activity after high temperature incubation of CcdB mutants was determined by SPR at room temperature.

| **Mutants** | **C_m_**  **(M)** | **ΔG⁰**  **(kcal.mol^-1^)** | **m_equi_**  **(kcal.mol^-1^M^-1^)** | **T_mRefold_**  **(°C)** | **T_mGdnCl_ (°C)** | **Residual activity (%)** | |
| --- | --- | --- | --- | --- | --- | --- | --- |
|  |  |  |  |  |  | **40 °C** | **80 °C** |
| **WT** | 2.8±0.1 | 21.1±0.7 | 4.97±0.5 | 53±0.5 | 52±0.8 | 24 | 0.8 |
| **E11R** | 2.3±0.2 | 18.0±1.8 | 4.55±0.5 | 55±0.4 | 55±0.5 | 1.2 | 0.6 |
| **S12G** | 3.0±0.3 | 21.9±1.4 | 4.90±0.2 | 55±0.9 | 55±1.4 | 61 | 3.7 |
| **V18W** | 0.8±0.7 | 11.0±1.8 | 4.00±1.2 | ^2^ | ^2^ | - | - |
| **V18W-S12G** | 1.3±0.1 | 13.5±0.8 | 4.87±0.2 | 45±0.4 | 43±0.9 | - | - |
| **V20F** | 0.3±0.1 | 8.6±1.8 | 4.06±0.2 | ^3^ | ^3^ | - | - |
| **V20F-S12G** | 1.0±0.4 | 12.1±1.4 | 4.88±0.2 | 43±1.2 | 43±0.6 | - | - |
| **L36A** | 1.5±0.4 | 13.9±0.3 | 4.45±1.4 | 45±1.4 | 46±0.9 | 11.7 | 0.4 |
| **L36A-E11R** | 2.4±0.4 | 18.9±1.2 | 4.50±0.5 | 51±0.8 | 51±0.4 | 34 | 2.4 |
| **L36A-S12G** | 2.4±0.3 | 18.8±0.9 | 4.82±0.7 | 52±1.2 | 51±0.6 | 47.6 | 9.1 |
| **L83S** | 1.6±0.1 | 14.4±1.7 | 4.43±0.8 | 43±0.2 | 41±1.8 | - | - |
| **L83S-E11R** | 2.1±0.2 | 18.3±3.2 | 4.73±0.5 | 54±0.1 | 53±0.6 | - | - |
| **L83S-S12G** | 2.3±0.9 | 18.6±1.3 | 4.70±0.9 | 55±0.9 | 55±0.6 | - | - |

^1^Reported standard errors are derived from two independent experiments, each performed in duplicates.

^2,3^ Could not be refolded in 1.5 M GdnCl. All the other proteins were refolded in 1.5 M GdnCl.

-Not determined.
