# Supplementary material for: Mechanistic insights into global suppressors of protein folding defects
Source: PLoS Genet. 2022 Aug 29;18(8):e1010334. doi: 10.1371/journal.pgen.1010334 (PMC9491731; doi:10.1371/journal.pgen.1010334)
Supplement: S3 Table — 1Reported standard errors are derived from two independent experiments, each performed in duplicates. (DOCX) [file pgen.1010334.s012.docx]

**S3_Table.** **Kinetic Parameters for refolding and unfolding of CcdB mutants measured under different buffer conditions at 25 °C, pH 8.4^1^ (Related to Fig 2).**

| **Mutant** | **[GdnCl]**  **(Refold/**  **Unfold) (M)** | **Refolding** | | | | | **Unfolding** | | |
| --- | --- | --- | --- | --- | --- | --- | --- | --- | --- |
|  |  | **Fast Phase** | | | **Slow Phase** | | **A0** | **A1** | **ku_1_ (s^-1^)** |
|  |  | **a0** | **a1** | **kf_1_ (s^-1^)** | **a2** | **kf_2_ (s^-1^)** |  |  |  |
| **WT** | **(1.5/3.5)** | 0.02±  0.02 | 0.55±  0.03 | 0.027  ±0.02 | 0.43±  0.03 | 0.002±  0.001 | 0.60±  0.03 | 0.40±  0.01 | 0.05±  0.05 |
| **E11R** | **(1.5/3.5)** | 0.14±  0.03 | 0.44±  0.08 | 0.080  ±0.01 | 0.42±  0.04 | 0.02±  0.0066 | 0.51±  0.04 | 0.49±  0.04 | 0.04±  0.08 |
| **S12G** | **(1.5/3.5)** | 0.03±  0.03 | 0.79±  0.02 | 0.041  ±0.05 | 0.18±  0.02 | 0.001±  0.003 | 0.44±  0.02 | 0.56±  0.03 | 0.03±  0.03 |
| **V18W** | **(0.5/2.0)** | 0.03±  0.06 | 0.52±  0.03 | 0.030  ±0.03 | 0.45±  0.06 | 0.003±  0.01 | 0.63±  0.09 | 0.37±  0.09 | 0.29±  0.03 |
| **V18W-S12G** | **(0.5/2.0)** | 0.20±  0.01 | 0.40±  0.08 | 0.095  ±0.13 | 0.40±  0.06 | 0.015±  0.044 | 0.46±  0.03 | 0.54±  0.03 | 0.10±  0.05 |
| **V20F** | **(0.5/2.0)** | 0.02±  0.05 | 0.71±  0.06 | 0.024  ±0.01 | 0.27±  0.06 | 0.002±  0.07 | 0.76±  0.01 | 0.24±  0.01 | 0.44±  0.08 |
| **V20F-S12G** | **(0.5/2.0)** | 0.02±  0.07 | 0.67±  0.15 | 0.104  ±0.09 | 0.31±  0.08 | 0.02±  0.002 | 0.66±  0.04 | 0.34±  0.03 | 0.16±  0.04 |
| **L36A** | **(1.0/3.5)** | 0.05±  0.09 | 0.55±  0.04 | 0.016  ±0.08 | 0.40±  0.08 | 0.001±  0.001 | 0.77±  0.08 | 0.27±  0.09 | 0.24±  0.02 |
| **L36A-E11R** | **(1.0/3.5)** | 0.10±  0.02 | 0.53±  0.03 | 0.046  ±0.02 | 0.36±  0.03 | 0.001±  0.001 | 0.61±  0.01 | 0.39±  0.01 | 0.17±  0.08 |
| **L36A-S12G** | **(1.0/3.5)** | 0.10±  0.01 | 0.54±  0.07 | 0.064  ±0.01 | 0.34±  0.05 | 0.007±  0.0066 | 0.64±  0.02 | 0.36±  0.02 | 0.07±  0.03 |
| **L83S** | **(1.0/3.5)** | 0.03±  0.01 | 0.62±  0.01 | 0.032  ±0.05 | 0.35±  0.02 | 0.004±  0.006 | 0.77±  0.03 | 0.23±  0.03 | 0.28±  0.09 |
| **L83S-E11R** | **(1.0/3.5)** | 0.62±0.04 | - | - | 0.38±  0.04 | 0.035±  0.001 | 0.72±  0.01 | 0.28±  0.02 | 0.10±  0.03 |
| **L83S-S12G** | **(1.0/3.5)** | 0.02±  0.07 | 0.62±  0.02 | 0.096  ±0.08 | 0.36±  0.01 | 0.012±  0.01 | 0.71±  0.06 | 0.29±  0.06 | 0.15±  0.08 |

^1^Reported standard errors are derived from two independent experiments, each performed in duplicates.
