# Supplementary material for: Mechanistic insights into global suppressors of protein folding defects
Source: PLoS Genet. 2022 Aug 29;18(8):e1010334. doi: 10.1371/journal.pgen.1010334 (PMC9491731; doi:10.1371/journal.pgen.1010334)
Supplement: S4 Table — 1Reported standard errors are derived from two independent experiments, each performed in duplicates. 2Reported rate constants are extrapolated to 0 M GdnCl for relative comparison among different mutants. 3 Fast phase of refolding couldn’t be captured. (DOCX) [file pgen.1010334.s013.docx]

**S4_Table.** **Relative Kinetic Parameters for *in vitro* Refolding and Unfolding of CcdB mutants extrapolated to zero denaturant^1^ (Related to Fig 2).**

| **Mutants** | **Refolding** | | | | **Unfolding** | |
| --- | --- | --- | --- | --- | --- | --- |
|  | **Fast Phase** | | **Slow Phase** | | **m_kinetic (U)_ value (M^-1^s^-1^)** | **rate constant^2^ (s^-1^)** |
|  | **m_kinetic_ _fast_ value (M^-1^s^-1^)** | **rate constant^2^ (s^-1^)** | **m_kinetic_ _slow_ value (M^-1^s^-1^)** | **rate constant^2^ (s^-1^)** |  |  |
| **WT** | 0.29±0.004 | 0.44±0.04 | 0.03±0.002 | 0.05±0.01 | 0.02±0.002 | 0.005±0.01 |
| **E11R** | 0.58±0.005 | 0.94±0.009 | 0.09±0.003 | 0.15±0.02 | 0.02±0.003 | 0.002±0.02 |
| **S12G** | 0.52±0.010 | 0.81±0.12 | 0.08±0.001 | 0.13±0.008 | 0.02±0.001 | 0.002±0.008 |
| **V18W** | 0.06±0.005 | 0.06±0.001 | 9e-3±0.003 | 7e-3±0.001 | 0.35±0.08 | 0.032±0.08 |
| **V18W-S12G** | 0.20±0.009 | 0.19±0.01 | 0.03±0.004 | 0.06±0.004 | 0.14±0.004 | 0.019±0.004 |
| **V20F** | 0.03±0.006 | 0.04±0.004 | 4e-3±0.002 | 4e-3±0.001 | 0.45±0.08 | 0.045±0.01 |
| **V20F-S12G** | 0.26±0.09 | 0.23±0.12 | 0.04±0.03 | 0.04±0.01 | 0.16±0.004 | 0.015±0.01 |
| **L36A** | 0.10±0.02 | 0.11±0.05 | 0.01±0.01 | 0.01±0.02 | 0.12±0.03 | 0.019±0.04 |
| **L36A-E11R** | 0.21±0.02 | 0.26±0.03 | 0.03±0.01 | 0.03±0.01 | 0.06±0.0001 | 0.011±0.003 |
| **L36A-S12G** | 0.39±0.007 | 0.44±0.01 | 0.06±0.002 | 0.06±0.04 | 0.05±0.001 | 0.010±0.001 |
| **L83S** | 0.13±0.02 | 0.17±0.03 | 0.02±0.002 | 0.02±0.003 | 0.11±0.003 | 0.012±0.02 |
| **L83S-E11R** | -^3^ | -^3^ | 0.10±0.003 | 0.16±0.01 | 0.04±0.0008 | 0.004±2e-3 |
| **L83S-S12G** | 0.50±0.005 | 0.60±0.057 | 0.09±0.003 | 0.10±0.004 | 0.06±0.02 | 0.006±0.001 |

^1^Reported standard errors are derived from two independent experiments, each performed in duplicates.

^2^Reported rate constants are extrapolated to 0 M GdnCl for relative comparison among different mutants.

^3^ Fast phase of refolding couldn’t be captured.
