# Supplementary material for: Mechanistic insights into global suppressors of protein folding defects
Source: PLoS Genet. 2022 Aug 29;18(8):e1010334. doi: 10.1371/journal.pgen.1010334 (PMC9491731; doi:10.1371/journal.pgen.1010334)
Supplement: S5 Table — Top: MIC and IC90 for ampicillin and cefotaxime from OD600 and plate data measurements respectively, thermodynamic stability parameters (Cm, ΔG0, mequi), apparent thermal stability (Tm), thermal stabilities of refolded proteins and native proteins in presence of 0.5 M GdnCl (TmRefold,, TmGdnCl), of different TEM-1 β-lactamase mutants1. Bottom: Kinetic parameters for refolding and unfolding of TEM-1 β-lactamase mutants measured in 0.5 M and 2.5 M GdnCl respectively carried out in 10 mM HEPES, 300 mM NaCl, 10% glycerol, pH 7.0, at 25°C1 (Related to Fig 3). 1Reported standard errors are derived from two independent experiments, each performed in duplicates. NT- No Transition: Not refolded in 0.5 M GdnCl. All the other proteins were refolded in 0.5 M GdnCl. (DOCX) [file pgen.1010334.s014.docx]

**S5_Table.** **Top:** **MIC and IC_90_ for ampicillin and cefotaxime from OD_600_ and plate data measurements, thermodynamic stability parameters (C_m_, ΔG⁰, m_equi_), apparent thermal stability (T_m_), thermal stabilities of refolded proteins and native proteins in presence of 0.5 M GdnCl (T_mRefold,_, T_mGdnCl_), of different TEM-1 β-lactamase mutants^1^. Bottom: Kinetic Parameters for refolding and unfolding of TEM-1 β-lactamase mutants measured in 0.5 M and 2.5 M GdnCl respectively carried out in 10 mM HEPES, 300 mM NaCl, 10% glycerol, pH 7.0, at 25 °C^1^ (Related to Fig 3).**

| **Mutants** | **Ampicillin**  **(µg/mL)** | | **Cefotaxime**  **(µg/mL)** | | | | **C_m_**  **(M)** | **ΔG⁰**  **(kcal.mol^-1^)** | **m_equi_**  **(kcal.mol^-1^M^-1^)** | | **T_m_**  **(°C)** | **T_mRefold_**  **(°C)** | **T_mGdnCl,_ (°C)** |
| --- | --- | --- | --- | --- | --- | --- | --- | --- | --- | --- | --- | --- | --- |
|  | **MIC** | **IC_90_** | **MIC** | | **IC_90_** | |  |  |  |  |  |  |  |
| **WT** | ≈3000 | 4000 | 16 | | 10 | | 1.2±0.1 | 2.9±0.5 | 2.4±0.1 | | 51±0.2 | 41±0.4 | 41±0.2 |
| **M182T** | ≈3000 | 4000 | 16 | | 15 | | 1.9±0.3 | 4.7±0.6 | 3.1±0.3 | | 57±0.1 | 47±0.3 | 48±0.5 |
| **M69I** | ≈800 | 1000 | 8 | | 6 | | 0.6±0.1 | 2.0±0.2 | 2.1±0.2 | | 47±0.3 | 31±0.1 | 32±0.4 |
| **M69I-M182T** | ≈800 | 1000 | 8 | | 6 | | 1.4±0.2 | 4.0±0.3 | 2.8±0.1 | | 54±0.2 | 44±0.2 | 44±0.3 |
| **L76N** | ≈50 | 20 | 2 | | <1 | | 0.5±0.1 | 1.1±0.1 | 1.8±0.2 | | 40±0.5 | NT | NT |
| **L76N-M182T** | ≈1500 | 1500 | 4 | | 4 | | 1.1±0.2 | 2.8±0.3 | 2.3±0.4 | | 48±0.3 | 39±0.1 | 39±0.5 |
| **Mutants** | **Refolding** | | | | | | | | **Unfolding** | | | | |
|  | **Fast Phase** | | | | | **Slow Phase** | | | **A0** | **A1** | | **ku_1_ (s^-1^)** | |
|  | **a0** | **a1** | | **kf_1_ (s^-1^)** | | **a2** | | **kf_2_ (s^-1^)** |  |  |  |  |  |
| **WT** | 0.01±  0.02 | 0.51±  0.03 | | 0.049  ±0.01 | | 0.48±  0.03 | | 0.004±  0.001 | 0.65±  0.03 | 0.35±0.01 | | 0.03±  0.003 | |
| **M182T** | 0.03±  0.03 | 0.53±  0.08 | | 0.19  ±0.01 | | 0.44±  0.04 | | 0.008±  0.001 | 0.56±  0.04 | 0.44±0.04 | | 0.02±  0.002 | |
| **M69I** | 0.03±  0.03 | 0.55±  0.02 | | 0.016  ±0.01 | | 0.42±  0.02 | | 0.003±  0.004 | 0.71±  0.02 | 0.29±0.03 | | 0.07±  0.003 | |
| **M69I-M182T** | 0.03±  0.06 | 0.52±  0.03 | | 0.11  ±0.02 | | 0.45±  0.06 | | 0.006±  0.001 | 0.65±  0.09 | 0.35±0.09 | | 0.03±  0.001 | |
| **L76N** | 0.02±  0.01 | 0.60±  0.08 | | 0.014  ±0.01 | | 0.38±  0.06 | | 0.002±  0.002 | 0.79±  0.03 | 0.21±0.03 | | 0.10±  0.004 | |
| **L76N-M182T** | 0.03±  0.05 | 0.68±  0.06 | | 0.023  ±0.02 | | 0.29±  0.06 | | 0.003±  0.001 | 0.68±  0.01 | 0.32±0.01 | | 0.06±  0.003 | |

^1^Reported standard errors are derived from two independent experiments, each performed in duplicates.

NT- No Transition: Not refolded in 0.5 M GdnCl. All the other proteins were refolded in 0.5 M GdnCl.
