# Supplementary material for: Mechanistic insights into global suppressors of protein folding defects
Source: PLoS Genet. 2022 Aug 29;18(8):e1010334. doi: 10.1371/journal.pgen.1010334 (PMC9491731; doi:10.1371/journal.pgen.1010334)
Supplement: S6 Table — Top: Fractional solubilities, thermodynamic stability parameters (Cm, ΔG0, mequi), apparent thermal stabilities (Tm), thermal stabilities of refolded and native proteins in the presence of 0.5 M Urea (TmRefold,,Tm,urea) determined by nanoDSF, of different p53 mutants1. Bottom: Kinetic parameters for refolding and unfolding of p53 mutants measured in 2 M and 4.4 M Urea respectively carried out in 50 mM NaH2PO4, 500 mM NaCl, 500 mM imidazole, 10 mM DTT, pH 7.2 at 15°C1 (Related to Fig 4). 1Reported standard errors are derived from two independent experiments, each performed in duplicates. ND–Not determined as the corresponding proteins could not be purified. (DOCX) [file pgen.1010334.s015.docx]

**S6_Table. Top: Fractional solubilities, thermodynamic stability parameters (C_m_, ΔG⁰, m_equi_), apparent thermal stabilities (T_m_), thermal stabilities of refolded and native proteins in the presence of 0.5 M Urea (T_mRefold,_,T_m,urea_) determined by nanoDSF, of different p53 mutants^1^. Bottom: Kinetic Parameters for refolding and unfolding of p53 mutants measured in 2 M and 4.4 M Urea respectively carried out in 50 mM NaH_2_PO_4_, 500 mM NaCl, 500 mM imidazole, 10 mM DTT, pH 7.2 at 15 °C^1^ (Related to Fig 4).**

| **Mutants** | **Fraction soluble** | | | **C_m_**  **(M)** | | **ΔG⁰**  **(kcal.mol^-1^)** | | **m_equi_**  **(kcal.mol^-1^M^-1^)** | | **T_m_**  **(°C)** | | **T_m,refold_**  **(°C)** | | **T_m,urea_ (°C)** | |
| --- | --- | --- | --- | --- | --- | --- | --- | --- | --- | --- | --- | --- | --- | --- | --- |
| **WT** | 0.4±0.05 | | | 2.8±0.02 | | 2.5±0.1 | | 0.9±0.01 | | 30±0.1 | | 29±0.1 | | 29±0.1 | |
| **N239Y** | 0.5±0.05 | | | 3.0±0.05 | | 2.6±0.2 | | 0.9±0.02 | | 32±0.1 | | 29±0.4 | | 30±0.1 | |
| **V143A** | 0.1±0.04 | | | ND | | ND | | ND | | ND | | ND | | ND | |
| **V143A-N239Y** | 0.3±0.02 | | | 3.1±0.1 | | 2.7±0.1 | | 0.9±0.002 | | 31±0.1 | | 29±0.1 | | 27±0.1 | |
| **V157F** | 0.1±0.05 | | | ND | | ND | | ND | | ND | | ND | | ND | |
| **V157F -N239Y** | 0.4±0.04 | | | 3.3±0.05 | | 2.9±0.1 | | 0.9±0.005 | | 33±0.1 | | 32±0.1 | | 32±0.1 | |
| **Mutants** | **Refolding** | | | | | | | **Unfolding** | | | | | | | |
|  | **Fast Phase** | | | | **Slow Phase** | | | **A0** | **A1** | | **ku_1_ (s^-1^)** | | **A2** | | **ku_2_ (s^-1^)** |
|  | **a0** | **a1** | **kf_1_ (s^-1^)** | | **a2** | | **kf_2_ (s^-1^)** |  |  |  |  |  |  |  |  |
| **WT** | 0.68± 0.01 | 0.14±  0.0004 | 0.024  ±0.0003 | | 0.18±  0.005 | | 0.0003±  0.00001 | 0.35±  0.01 | 0.53±  0.01 | | 0.046±  0.0005 | | 0.12±  0.002 | | 0.003± 0.00005 |
| **N239Y** | 0.73±  0.002 | 0.14±  0.0004 | 0.037  ±0.0003 | | 0.13±  0.007 | | 0.006±  0.005 | 0.32±  0.009 | 0.56±  0.009 | | 0.037±  0.0006 | | 0.12±  0.002 | | 0.0025±  0.00007 |
| **V143A N239Y** | 0.86±  0.003 | 0.05±  0.0003 | 0.023  ±0.003 | | 0.09±  0.005 | | 0.003±  0.006 | 0.40±  0.01 | 0.37±  0.01 | | 0.028±  0.001 | | 0.23±  0.007 | | 0.0017±  0.0005 |
| **V157F N239Y** | 0.87±  0.002 | 0.02± 0.001 | 0.083  ±0.04 | | 0.11±  0.002 | | 0.0004±  0.00001 | 0.49±  0.003 | 0.23±  0.003 | | 0.023±  0.0015 | | 0.28±  0.003 | | 0.0019±  0.00006 |

^1^Reported standard errors are derived from two independent experiments, each performed in duplicates.

ND – Not determined as the corresponding proteins could not be purified.
