# Supplementary material for: Mechanistic insights into global suppressors of protein folding defects
Source: PLoS Genet. 2022 Aug 29;18(8):e1010334. doi: 10.1371/journal.pgen.1010334 (PMC9491731; doi:10.1371/journal.pgen.1010334)
Supplement: S7 Table — 1Reported standard errors are derived from two independent experiments, each performed in duplicates. WT values are taken from S5 Table. (DOCX) [file pgen.1010334.s016.docx]

**S7_Table.** **Thermodynamic parameters (C_m_, ΔG⁰, m_equi_), determined by nanoDSF, of CcdB mutants^1^ (Related to Fig 5).**

| **Mutants** | **C_m_ (M)** | **ΔG⁰ (kcal.mol^-1^)** | **m_equi_ (kcal.mol^-1^M^-1^)** |
| --- | --- | --- | --- |
|  |  |  |  |
| **CcdB WT** | 2.8±0.1 | 21.1±0.7 | 4.97±0.5 |
| **CcdB Y8D** | 3.1±0.3 | 22.7±1.2 | 5.00±0.1 |
| **CcdB V46L** | 3.2±0.3 | 23.2±1.4 | 5.01±0.3 |
| **CcdB S60E** | 3.5±0.5 | 24.8±1.1 | 5.03±0.2 |

^1^Reported standard errors are derived from two independent experiments, each performed in duplicates. WT values are taken from S5_Table.
