# Supplementary material for: Mechanistic insights into global suppressors of protein folding defects
Source: PLoS Genet. 2022 Aug 29;18(8):e1010334. doi: 10.1371/journal.pgen.1010334 (PMC9491731; doi:10.1371/journal.pgen.1010334)
Supplement: S8 Table — Top: Kinetic parameters for refolding and unfolding of stabilised CcdB mutants measured in 2 M and 4.5 M GdnCl respectively, carried out in 200 mM HEPES, pH 8.4 at 25°C1. Bottom: Kinetic parameters for refolding and unfolding of stabilised mRBD mutants in 0.5 M and 3 M GdnCl respectively, carried out in 1X PBS, pH 7.0 at 25°C1 (Related to Fig 5). 1Reported standard errors are derived from two independent experiments, each performed in duplicates. (DOCX) [file pgen.1010334.s017.docx]

**S8_Table. Top: Kinetic Parameters for refolding and unfolding of stabilised CcdB mutants measured in 2 M and 4.5 M GdnCl respectively, carried out in** **200 mM HEPES, pH 8.4 at 25 °C^1^. Bottom: Kinetic Parameters for refolding and unfolding of stabilised mRBD mutants in 0.5 M and 3 M GdnCl respectively, carried out in 1X PBS, pH 7.0 at 25 °C^1^ (Related to Fig 5).**

| **Mutants** | **Refolding (2 M)** | | | | | **Unfolding (4.5 M)** | | |
| --- | --- | --- | --- | --- | --- | --- | --- | --- |
|  | **Fast Phase** | | | **Slow Phase** | | **A0** | **A1** | **ku_1_ (s^-1^)** |
|  | **a0** | **a1** | **kf_1_ (s^-1^)** | **a2** | **kf_2_ (s^-1^)** |  |  |  |
| **CcdB WT** | 0.03±  0.01 | 0.50±  0.02 | 0.020  ±0.01 | 0.47±  0.02 | 0.001±  0.001 | 0.73±  0.01 | 0.27±0.02 | 0.060±  0.05 |
| **CcdB Y8D** | 0.01±  0.02 | 0.51±  0.03 | 0.06  ±0.01 | 0.48±  0.03 | 0.018±  0.001 | 0.58±  0.03 | 0.42±0.01 | 0.027±  0.003 |
| **CcdB V46L** | 0.03±  0.03 | 0.53±  0.08 | 0.10  ±0.01 | 0.44±  0.04 | 0.026±  0.001 | 0.55±  0.04 | 0.45±0.04 | 0.025±  0.002 |
| **CcdB S60E** | 0.03±  0.03 | 0.55±  0.02 | 0.13  ±0.01 | 0.42±  0.02 | 0.028±  0.004 | 0.52±  0.02 | 0.48±0.03 | 0.020±  0.003 |
| **Mutants** | **Refolding (0.5 M)** | | | **Unfolding (3 M)** | | | | |
|  | **a0** | **a1** | **kf_1_** **(s^-1^)** | **Fast Phase** | | | **Slow Phase** | |
|  |  |  |  | **A0** | **A1** | **ku_1_** **(s^-1^)** | **A2** | **ku_2_** **(s^-1^)** |
| **mRBD**  **WT** | 0.20±  0.01 | 0.80±  0.02 | 0.015  ±0.003 | 0.58±  0.02 | 0.28±  0.03 | 0.051  ±0.002 | 0.14±  0.03 | 0.002±  0.0005 |
| **mRBD D389E** | 0.26±  0.03 | 0.74±  0.01 | 0.038  ±0.004 | 0.42±  0.03 | 0.48±  0.08 | 0.016  ±0.003 | 0.10±  0.04 | 0.001±  0.0006 |
| **mRBD L390M** | 0.25±  0.02 | 0.75±  0.03 | 0.044  ±0.002 | 0.43±  0.03 | 0.47±  0.08 | 0.014  ±0.001 | 0.12±  0.04 | 0.001±  0.001 |
| **mRBD P527I** | 0.35±  0.02 | 0.65±  0.01 | 0.103  ±0.01 | 0.33±  0.03 | 0.52±  0.02 | 0.013  ±0.002 | 0.15±  0.02 | 0.001±  0.0007 |

^1^Reported standard errors are derived from two independent experiments, each performed in duplicates.
