# Supplementary material for: Mechanistic insights into global suppressors of protein folding defects
Source: PLoS Genet. 2022 Aug 29;18(8):e1010334. doi: 10.1371/journal.pgen.1010334 (PMC9491731; doi:10.1371/journal.pgen.1010334)
Supplement: S9 Table — Top: Thermodynamic parameters (Cm, ΔG0, mequi), apparent thermal stability (Tm), thermal stabilities of refolded and native proteins in presence of 1.5 M GdnCl (TmRefold, TmGdnCl). Bottom: Kinetic parameters for refolding and unfolding of CcdB WT, M32T, L42E and S43T in 1.5 M and 3.5 M GdnCl respectively carried out in 200 mM HEPES, pH 8.4 at 25°C1 (Related to Fig 7). 1Reported standard errors are derived from two independent experiments, each performed in duplicates. WT values of thermodynamic parameters are taken from S5 Table and kinetic parameters are taken from S2 Table. (DOCX) [file pgen.1010334.s018.docx]

**S9_Table. Top: Thermodynamic parameters (C_m_, ΔG⁰, m_equi_), apparent thermal stability (T_m_), thermal stabilities of refolded and native proteins in presence of 1.5 M GdnCl (T_mRefold_, T_mGdnCl_). Bottom: Kinetic parameters for refolding and unfolding of CcdB WT M32T, L42E and S43T in 1.5 M and 3.5 M GdnCl respectively of CcdB WT and L42E carried out in 200 mM HEPES, pH 8.4 at 25 °C^1^ (Related to Fig 7).**

| **Mutants** | **C_m_**  **(M)** | | **ΔG⁰**  **(kcal.mol^-1^)** | | **m_equi_**  **(kcal.mol^-1^M^-1^)** | | | **T_m_**  **(°C)** | **T_mRefold_**  **(°C)** | | **T_mGdnCl_**  **(°C)** | |
| --- | --- | --- | --- | --- | --- | --- | --- | --- | --- | --- | --- | --- |
|  |  |  |  |  |  |  |  |  |  |  |  |  |
| **WT** | 2.8±0.1 | | 21.1±0.7 | | 4.97±0.5 | | | 66.0±0.8 | 53.0±0.5 | | 52.0±0.8 | |
| **M32T** | 2.4±0.2 | | 18.2±0.5 | | 4.59±0.2 | | | 58.9±0.3 | 44.5±0.2 | | 44.3±0.1 | |
| **L42E** | 3.1±0.1 | | 21.5±0.03 | | 4.60±0.3 | | | 67.4±0.2 | 54.9±0.1 | | 55.1±0.1 | |
| **S43T** | 3.2±0.2 | | 21.7±0.2 | | 4.51±0.4 | | | 68.2±0.1 | 56.6±0.3 | | 56.3±0.1 | |
| **Mutants** | **Refolding** | | | | | | | | **Unfolding** | | | |
|  | **Fast Phase** | | | | | **Slow Phase** | | | **A0** | **A1** | | **ku_1_ (s^-1^)** |
|  | **a0** | **a1** | | **kf_1_ (s^-1^)** | | **a2** | **kf_2_ (s^-1^)** | |  |  |  |  |
| **WT** | 0.02±  0.02 | 0.55±  0.03 | | 0.027  ±0.02 | | 0.43±  0.03 | 0.002±  0.001 | | 0.60±  0.03 | 0.40±  0.01 | | 0.050±  0.05 |
| **M32T** | 0.04±  0.02 | 0.68±  0.02 | | 0.029  ±0.01 | | 0.28±  0.01 | 0.001±  0.0005 | | 0.57±  0.01 | 0.43±  0.02 | | 0.055±  0.01 |
| **L42E** | 0.03±  0.001 | 0.67±  0.01 | | 0.023  ±0.01 | | 0.33±  0.01 | 0.002±  0.001 | | 0.63±  0.02 | 0.37±0.01 | | 0.054±  0.001 |
| **S43T** | 0.02±  0.01 | 0.63±  0.02 | | 0.028  ±0.02 | | 0.35±  0.03 | 0.002±  0.001 | | 0.59±  0.02 | 0.41±  0.02 | | 0.058±  0.05 |

^1^Reported standard errors are derived from two independent experiments, each performed in duplicates. WT values of thermodynamic parameters are taken from S5_Table and kinetic parameters are taken from S2_Table.
