# Supplementary material for: Mechanistic insights into global suppressors of protein folding defects
Source: PLoS Genet. 2022 Aug 29;18(8):e1010334. doi: 10.1371/journal.pgen.1010334 (PMC9491731; doi:10.1371/journal.pgen.1010334)
Supplement: S10 Table — *: Values within brackets are for highest resolution shell. (DOCX) [file pgen.1010334.s019.docx]

**S10_Table.** **Data collection and refinement statistics for CcdB mutants (Related to Fig 6).**

| **Data collection** | **S12G** | **V46L** | **S60E** |
| --- | --- | --- | --- |
| Space group | I2 | C2 | C2 |
| a, b, c (Å) | 35.57, 36.53, 67.53 | 75.10, 36.76, 35.91 | 74.81, 36.64, 35.67 |
| α, β, γ (°) | 90, 93.69, 90 | 90, 115.19, 90 | 90, 114.97, 90 |
| Wavelength (Å) | 1.5418 | 1.0358 | 1.5418 |
| Resolution range (Å) | 33.69 – 1.63 (1.72 – 1.63) | 33.98-1.35 (1.43-1.35) | 10.00-1.93 (2.03-1.93) |
| Total No. of reflections | 48336 (6679) | 114256 (14182) | 22462 (2937) |
| No. of unique reflections | 9822 (1360) | 19433 (2799) | 6557 (890) |
| Multiplicity | 4.9 (4.9) | 5.9 (5.1) | 3.4 (3.3) |
| Completeness (%) | 90.6 (86.9) | 100 (100) | 97.5 (91.6) |
| 〈I/σ(I)〉 | 9.8 (4.8) | 11.6 (3.7) | 9.6 (3.8) |
| Wilson B factor (Å^2^) | 11.66 | 5.78 | 8.6 |
| *R*p.i.m. | 0.041 (0.097) | 0.043 (0.141) | 0.081 (0.268) |
| *R*merge | 0.083 (0.195) | 0.098 (0.288) | 0.130 (0.424) |
| *R*meas | 0.093 (0.218) | 0.108 (0.322) | 0.154 (0.504) |
| CC1/2 | 0.995 (0.967) | 0.994 (0.928) | 0.99 (0.801) |
| **Refinement** | | |  |
| *R*work (%) | 22.8 | 16.2 | 17.0 |
| *R*free (%) | 26.9 | 18.4 | 21.4 |
| **RMSD** | | | |
| Bond (Å) | 0.0072 | 0.0138 | 0.0051 |
| Angle (°) | 1.4844 | 1.9222 | 1.3247 |
| **Ramachandran plot** |  |  |  |
| Ramachandran favoured (%) | 98 | 100.0 | 99 |
| Ramachandran allowed (%) | 2 | 0 | 1 |
| Ramachandran outliers (%) | 0 | 0 | 0 |
| **Average B factor (Å2)** | | | |
| Overall | 18.85 | 11.19 | 11.37 |
| Protein | 18.05 | 9.13 | 10.57 |
| Solvent | 25.77 | 21.25 | 18.08 |
| Other | 17.70 | 8.71 | 11.53 |
| **Total no of atoms** | |  |  |
| Protein atoms | 816 | 814 | 820 |
| Solvent | 95 | 167 | 98 |
| Other | 2 Cl^-^ | 2 Cl^-^ | 2 Cl^-^ |
| Z | 1 | 1 | 1 |

* : values within brackets are for highest resolution shell.
