# Supplementary material for: Mechanistic insights into global suppressors of protein folding defects
Source: PLoS Genet. 2022 Aug 29;18(8):e1010334. doi: 10.1371/journal.pgen.1010334 (PMC9491731; doi:10.1371/journal.pgen.1010334)
Supplement: S11 Table — ΔΔG°, ΔTm, a0, kf1, kf2, A0, ku1, ku2 represent the difference in the Gibbs free energy, difference in melting temperature, fold change of amplitude of burst phase of refolding, rate constant of fast phase of refolding, rate constant of slow phase of refolding, amplitude of burst phase of unfolding, rate constant of fast phase of unfolding, rate constant of slow phase of unfolding respectively. (DOCX) [file pgen.1010334.s020.docx]

**S11_Table.** **Average difference and fold change of various thermodynamic and kinetic parameters (Mean±SEM) respectively for the suppressor mutations averaged over data for mutants of CcdB, TEM-1 β-lactamase and mRBD** **(Related to Fig 8)**.

| **Ratio** | **Difference in thermodynamic parameter** | | **Refolding kinetic parameter ratio** | | | **Unfolding kinetic parameter ratio** | | |
| --- | --- | --- | --- | --- | --- | --- | --- | --- |
|  |  |  | **Fast** | | **Slow** | **Fast** | | **Slow** |
|  | **ΔΔG°**  **(kcal.mol^-1^)** | **ΔT_m_**  **(°C)** | **a0** | **kf_1_** | **kf_2_** | **A0** | **ku_1_** | **ku_2_** |
| WT/  Suppressor | 2.2±0.8 | 1.9±1.1 | 2.1±0.6 | 3.3±0.5 | 9.8±2.6 | 0.8±0.03 | 0.5±0.1 | 0.6±0.1 |
| PIM/  PIM-Suppressor | 3.5±0.5 | 8.1±1.0 | 4.5±2.4 | 3.7±0.6 | 4.8±1.2 | 0.9±0.03 | 0.45±0.1 | - |

ΔΔG°, ΔT_m_, a0, kf_1_, kf_2_, A0, ku_1_, ku_2_ represents the difference in the Gibbs free energy, melting temperature, fold change of amplitude of fast phase of refolding, rate constant of fast phase of refolding, rate constant of slow phase of refolding, amplitude of fast phase of unfolding, rate constant of fast phase of unfolding, rate constant of slow phase of unfolding respectively.
