# Supplementary material for: Mechanistic insights into global suppressors of protein folding defects
Source: PLoS Genet. 2022 Aug 29;18(8):e1010334. doi: 10.1371/journal.pgen.1010334 (PMC9491731; doi:10.1371/journal.pgen.1010334)
Supplement: S12 Table — n.a. not applicable as this phase is absent. a0 –amplitude of burst phase of refolding process. kf1, kf2 –refolding rate constants of fast and slow phases respectively. A0 – amplitude of burst phase of unfolding process. ku1, ku2 – unfolding rate constants of fast and slow phases respectively. (DOCX) [file pgen.1010334.s021.docx]

**S12_Table.** **Average difference and fold change of various thermodynamic and kinetic parameters (Mean±SEM) respectively for the suppressor mutations in the individual proteins CcdB, TEM-1 β-lactamase and mRBD** **(Related to S8 Fig)**.

| **Protein** | **Refolding kinetic parameter ratio** | | | **Unfolding kinetic parameter ratio** | | |
| --- | --- | --- | --- | --- | --- | --- |
|  | **Fast** | | **Slow** |  |  |  |
|  | **a0** | **kf_1_** | **kf_2_** | **A0** | **ku_1_** | **ku_2_** |
| CcdB | 4.0±1.6 | 3.0±0.3 | 6.8±1.4 | 0.8±0.1 | 0.5±0.1 | n.a. |
| TEM-1 β-lactamase | 1.8±0.6 | 4.2±1.5 | 1.8±0.2 | 0.9±0.1 | 0.6±0.1 | n.a. |
| mRBD | 1.4±0.2 | 4.1±1.4 | n.a. | 0.7±0.1 | 0.3±0.1 | 0.5±0.01 |

n.a. not applicable as this phase is absent. a0 – amplitude of burst phase of refolding process. kf_1_, kf_2_ – refolding rate constants of fast and slow phases respectively.
