# Supplementary material for: Mechanistic insights into global suppressors of protein folding defects
Source: PLoS Genet. 2022 Aug 29;18(8):e1010334. doi: 10.1371/journal.pgen.1010334 (PMC9491731; doi:10.1371/journal.pgen.1010334)
Supplement: S13 Table — 1A: Chain ID, SC: Side Chain, MC: Main Chain (DOCX) [file pgen.1010334.s022.docx]

**S13_Table.** **Additional Hydrogen bonds present in structures of suppressor mutants but absent in WT, calculated using HBPLUS [1]** **(Related to S9 Fig)**.

| **Additional interactions in mutant CcdB-S12G^1^** | **Additional interactions in mutant CcdB-V46L^1^** | **Additional interactions in mutant CcdB-S60E^1^** |
| --- | --- | --- |
| A:4:LYS--HBOND:  SC_SC--A:79:GLU | A:4:LYS--HBOND:  SC_SC--A:79:GLU | A:4:LYS--HBOND:  SC_SC--A:79:GLU |
| A:7:THR-- HBOND:  MC_SC--A:8:TYR | A:39:ALA--HBOND:  MC_MC--A:60:SER | A:7:THR--HBOND:  SC_MC--A:15:ARG |
| A:8:TYR--HBOND:  MC_MC--A:15:ARG | A:40:ARG--HBOND:  MC_SC--A:38:SER | A:27:THR--HBOND:  SC_MC--A:30:ARG |
| A:14:TYR--HBOND:  MC_SC--A:67:ASP | A:42:LEU-- HBOND:  MC_MC--A:60:SER | A:30:ARG--HBOND:  SC_SC--A:70:SER |
| A:31:ARG--HBOND:  SC_MC--A:22:SER | A:62:ARG--HBOND:  SC_MC--A:46:LEU (2) | A:48:ARG--HBOND:  SC_MC--A:44:ASP |
| A40:ARG--HBOND:  MC_SC--A:38:SER | A:65:THR--HBOND:  MC_MC--A:63:MET | A:49:GLU--HBOND:  MC_SC--A:47:SER |
| A:42:LEU--HBOND:  MC_MC--A:39:ALA |  | A:55:HIS--HBOND:  SC_SC--A:60:GLU |
| A:47:SER--HBOND:  SC_SC--A:49:GLU |  | A:62:ARG--HBOND:  SC_MC--A:44:ASP |
| A:62:ARG--HBOND:  SC_MC--A:46:VAL (2) |  | A:62:ARG--HBOND:  SC_MC--A:46:VAL (2) |
| A:86:ARG--HBOND:  SC_MC--A:53:VAL |  | A:86:ARG--HBOND:  SC_MC--A:53:VAL |
| A:90:ILE--HBOND:  MC_MC--A:86:ARG |  |  |
| A:97:MET--HBOND:  MC_MC--A:94:ILE |  |  |
| A:98:PHE--HBOND:  MC_MC--A:94:ILE |  |  |

^1^**A:** Chain ID**, SC:** Side Chain**, MC:** Main Chain

1. McDonald IK, Thornton JM. Satisfying hydrogen bonding potential in proteins. J Mol Biol. 1994;238: 777–793. doi:10.1006/jmbi.1994.1334
