# Supplementary material for: Mechanistic insights into global suppressors of protein folding defects
Source: PLoS Genet. 2022 Aug 29;18(8):e1010334. doi: 10.1371/journal.pgen.1010334 (PMC9491731; doi:10.1371/journal.pgen.1010334)
Supplement: S14 Table — Only residues with |ΔASA|>10Å2 are shown. 1ΔASA = (Total side chain ASA)Mutant – (Total side chain ASA)WT. (DOCX) [file pgen.1010334.s023.docx]

**S14_Table.** **Difference in Accessible Surface Area (ΔASA) between mutant and WT CcdB structures calculated using NACCESS [1]** **(Related to S9 Fig)**. Only residues with ⎢ΔASA⎢>10Å^2^ are shown.

| **S12G** | | | | **V46L** | | | | **S60E** | | | |
| --- | --- | --- | --- | --- | --- | --- | --- | --- | --- | --- | --- |
| **Res** | **Chain**  **ID** | **No.** | **ΔASA^1^**  **≥\|10\|Å^2^** | **Res** | **Chain**  **ID** | **No.** | **ΔASA^1^**  **≥\|10\|Å^2^** | **Res** | **Chain**  **ID** | **No.** | **ΔASA^1^**  **≥\|10\|Å^2^** |
| **GLY** | A | 12 | 54.5 | **MET** | A | 1 | -11.2 | **MET** | A | 1 | -11.4 |
| **ARG** | A | 13 | -26.6 | **ARG** | A | 13 | -25.1 | **GLY** | A | 29 | 11.6 |
| **ALA** | A | 37 | 17.8 | **TYR** | A | 14 | 11.8 | **SER** | A | 38 | -10.2 |
| **LEU** | A | 41 | -46.7 | **ARG** | A | 40 | -61.2 | **SER** | A | 43 | 13.1 |
| **LEU** | A | 42 | -18.3 | **LEU** | A | 41 | -47.3 | **ASP** | A | 44 | -36.8 |
| **ASP** | A | 44 | -28.9 | **SER** | A | 43 | -18.1 | **LYS** | A | 45 | -17.3 |
| **LYS** | A | 45 | -46.3 | **ASP** | A | 44 | -65.3 | **VAL** | A | 53 | -14.1 |
|  |  |  |  | **LYS** | A | 45 | -69.1 | **GLU** | A | 59 | 17.0 |
|  |  |  |  | **LEU** | A | 46 | -16.7 | **GLU** | A | 60 | 18.4 |
|  |  |  |  | **ARG** | A | 48 | -10.5 | **ASN** | A | 92 | -12.0 |
|  |  |  |  | **GLU** | A | 59 | 50.5 | **GLY** | A | 100 | -41.8 |
|  |  |  |  | **ILE** | A | 101 | -67.5 |  |  |  |  |

^1^ **ΔASA:** (Total side chain ASA)_mutant_-(Total side chain ASA)_WT_

1. Hubbard, S.J. and Thornton JM. NACCESS. In: “NACCESS” Computer Program, Department of Biochemistry and Molecular Biology, University College London, UK. Retrieved from http://www.bioinf.manchester.ac.uk/naccess/. 1993.
