# Supplementary material for: Mechanistic insights into global suppressors of protein folding defects
Source: PLoS Genet. 2022 Aug 29;18(8):e1010334. doi: 10.1371/journal.pgen.1010334 (PMC9491731; doi:10.1371/journal.pgen.1010334)
Supplement: Attachment — Submitted filename: Response to reviewers.docx [file pgen.1010334.s024.docx]

Dear Dr Varadarajan,

Thank you very much for submitting your Research Article entitled 'Mechanistic insights into global suppressors of protein folding defects' to PLOS Genetics.

The manuscript was fully evaluated at the editorial level and by independent peer reviewers. The reviewers appreciated the attention to an important topic but identified some concerns that we ask you address in a revised manuscript.

We therefore ask you to modify the manuscript according to the review recommendations. Your revisions should address the specific points made by each reviewer.

In addition we ask that you:

1) Provide a detailed list of your responses to the review comments and a description of the changes you have made in the manuscript.

2) Upload a Striking Image with a corresponding caption to accompany your manuscript if one is available (either a new image or an existing one from within your manuscript). If this image is judged to be suitable, it may be featured on our website. Images should ideally be high resolution, eye-catching, single panel square images. For examples, please browse our [archive](https://journals.plos.org/plosgenetics/volume). If your image is from someone other than yourself, please ensure that the artist has read and agreed to the terms and conditions of the Creative Commons Attribution License. Note: we cannot publish copyrighted images.

We hope to receive your revised manuscript within the next 30 days. If you anticipate any delay in its return, we would ask you to let us know the expected resubmission date by email to plosgenetics@plos.org.
If present, accompanying reviewer attachments should be included with this email; please notify the journal office if any appear to be missing. They will also be available for download from the link below. You can use this link to log into the system when you are ready to submit a revised version, having first consulted our [Submission Checklist](http://journals.plos.org/plosgenetics/s/submit-now#loc-submission-checklist).

While revising your submission, please upload your figure files to the [Preflight Analysis and Conversion Engine](http://pace.apexcovantage.com/) (PACE) digital diagnostic tool. PACE helps ensure that figures meet PLOS requirements. To use PACE, you must first register as a user. Then, login and navigate to the UPLOAD tab, where you will find detailed instructions on how to use the tool. If you encounter any issues or have any questions when using PACE, please email us at figures@plos.org.
Please be aware that our [data availability policy](http://journals.plos.org/plosgenetics/s/data-availability) requires that all numerical data underlying graphs or summary statistics are included with the submission, and you will need to provide this upon resubmission if not already present. In addition, we do not permit the inclusion of phrases such as "data not shown" or "unpublished results" in manuscripts. All points should be backed up by data provided with the submission.

To enhance the reproducibility of your results, we recommend that you deposit your laboratory protocols in protocols.io, where a protocol can be assigned its own identifier (DOI) such that it can be cited independently in the future. Additionally, PLOS ONE offers an option to publish peer-reviewed clinical study protocols. Read more information on sharing protocols at

<https://plos.org/protocols?utm_medium=editorial-email&utm_source=authorletters&utm_campaign=protocols>

Please review your reference list to ensure that it is complete and correct. If you have cited papers that have been retracted, please include the rationale for doing so in the manuscript text, or remove these references and replace them with relevant current references. Any changes to the reference list should be mentioned in the rebuttal letter that accompanies your revised manuscript. If you need to cite a retracted article, indicate the article’s retracted status in the References list and also include a citation and full reference for the retraction notice.

PLOS has incorporated [Similarity Check](http://www.crossref.org/crosscheck.html), powered by iThenticate, into its journal-wide submission system in order to screen submitted content for originality before publication. Each PLOS journal undertakes screening on a proportion of submitted articles. You will be contacted if needed following the screening process.

To resubmit, you will need to go to the link below and 'Revise Submission' in the 'Submissions Needing Revision' folder.

********
Please let us know if you have any questions while making these revisions.

Yours sincerely,

Diarmaid Hughes

Associate Editor

PLOS Genetics

Josep Casadesús
Section Editor: Prokaryotic Genetics
PLOS Genetics

**Reviewer's Responses to Questions**

**Comments to the Authors:**

**Please note here if the review is uploaded as an attachment.**

**Reviewer #1:**

**This manuscript describes the suppressor effects of second mutations on the defect of functions of proteins by first mutation. The authors performed various experiments including equilibrium and kinetics analyses of protein folding and derived conclusions that the significant factor is a refolding rate, and the stability of the native structure of a protein is not important. The topic authors treated is interesting and significant in terms of the evolution of protein functions. Thus, I feel that this manuscript can be basically published but the following points should be reconsidered before publication.**

*Response:* We thank the reviewer for providing their valuable suggestions to improve the manuscript.

**I think that the less significance of stability of a native structure for suppressor effects is plausible. But, the significance of refolding rate for suppressor effects is difficult to understand. What process of refolding is significant? The authors should discuss this point. Of course, the definite factor of this phenomenon may be difficult to demonstrate at this stage, but at least the authors should speculate this point.**

*Response:* Proteins belonging to varied classes show different refolding transition kinetics, wherein mRBD (WT and mutants) shows monophasic refolding, whereas mutants and WT proteins of CcdB, DNA binding domain of p53 and TEM-1 β-lactamase show biphasic refolding. We have now listed increments in thermodynamic stability associated with the suppressor mutation both in the presence and absence of the PIM in Table S11. Additionally, the corresponding refolding and unfolding kinetic parameter ratios are also shown in the same Table. The data clearly demonstrate that apparent effects of the suppressor mutation are larger in the context of the PIM than in the WT protein. In addition, effects of the suppressor mutation on refolding rate parameters (both burst phase amplitude as well as refolding rate constants) are larger than corresponding changes in unfolding kinetic parameters. Thus, all aspects of refolding are affected, albeit to different extents in different proteins. In most cases, folding is non-two state, so it is difficult to interpret the data in terms of transition state stabilization as multiple transition states are involved. In simplistic terms, destabilizing mutations at buried sites typically slow down the folding process *in vitro*, we speculate that this also holds true *in vivo*. In the crowded environment of the cell this might facilitate off-pathway aggregation. Suppressor mutations that accelerate refolding can counteract this, enhancing the yield of properly folded, functional protein *in vivo*.

We have included these points in middle para of page 23 of the “Discussion” section and in Figure 8, Supplementary Figure S8 and Supplementary Tables S11, S12.

**It looks like that the native state of a suppressor mutant is less stable but its refolding rate becomes large as shown in the results of TEM-1 b lactamase. How these phenomena can be interpreted in terms of mutations? Which mutations make the native state unstable and which mutations stabilize the folding transition state? The authors should give some speculations. The similar analyses should be given for other proteins.**

*Response:* For all the mutant variants of all the proteins used in the study, we have seen that the native state of a suppressor mutant is not destabilised relative to its wild type counterpart, except for the functionally active CcdB E11R, a charge reversal substitution, and M32T which is polar substitution at a dimer interface resulting in a small amount of thermodynamic destabilisation relative to wild type (Supplementary Table S1, S2, S9). In most cases the thermodynamic as well as kinetic stability of the protein increases upon introduction of a suppressor mutation. However, as discussed above, kinetic effects are larger and more consistent and effects on refolding are larger than on unfolding, consistent with native state stabilization. Previous studies have reported that the M182T suppressor mutant in TEM-1 β-lactamase stabilises the enzyme by recruiting newly formed hydrogen bonds mediated by threonine182 and adjacent water molecules (Wang *et al*, 2002). Consistent with this earlier work, we also find that the M182T mutation results in significant stabilization of the WT protein (see Figure 3E, F) as well as large enhancement in the refolding rate parameters and a small decrease in the unfolding rate constant. Previous reports have also shown that core-engineered mutations like M69I and L76N destabilise the native state of the lactamase enzyme (Sideraki *et al*, 2001), similar to the low thermodynamic stabilities observed for these inactive mutants in our thermal and chemical denaturation assays (Supplementary Table S5). Addition of M182T suppressor mutation to these inactive mutants stabilises their folded states and restores their functional defects (Supplementary Table S5).

Similarly, for the DNA-binding domain of p53, V143A and V157F are core mutations that destabilise the native state of the protein and lead to low expression of soluble functional protein levels (Supplementary Table S6). V143A causes perturbations in almost all the residues of the β-sandwich and DNA-binding surface (Wong *et al*, 1999). V157F, one of the strongest destabilising oncogenic mutant, causes side-chain rearrangements in the core of the protein (Wallentine *et al*, 2013). The N239Y suppressor mutant, stabilises the native state of the protein by introducing new hydrophobic contacts and hydrogen-bonds with water molecules, which were absent in the wild type protein (Wallentine *et al*, 2013). It can, therefore, be hypothesised that both the destabilised mutants, V143A and V157F, could be functionally rescued and stabilised by the suppressor by stabilisation of their folded states upon introduction of alternative favourable interactions by Y239 in the protein.

For CcdB, in the present study, in the various crystal structures of CcdB mutants, for suppressors S12G, V46L and S60E there is formation of novel hydrogen-bonds with water molecules or adjacent residues, that were absent in the wild type protein. These are likely responsible for stabilisation of the native state of the protein by these suppressors (lines 349 – 384 in the Results section). These points have now been included in pages 18-20, in the Discussion section.

**Minor point;**

**In page 16, line 407, should “Figure 7G” be “Figure 7D”?**

**In page 16, line 410, should “Figure 7G” be “Figure 7E”?**

*Response:* We thank the reviewer for pointing this out. We have now made the necessary changes.

**In page 18, line 467, the conformation of from residue 9 to 15 (9KRESRYR15) should be presented in a figure.**

*Response:* We have now included this in Supplementary Figure S7.

**Reviewer #2:** **This manuscript describes a remarkably thorough characterization of global suppressors in CcdB, (a toxin involved in the maintenance of F-plasmid in Escherichia coli) together with three other well-studied proteins. CcdB (Controller of Cell Death protein B) is particularly well characterized using a protocol that selects for a population of inactive mutants and then selects for global suppressors within this population.**

**Mutations are identified that stabilize the WT protein but fail to act as global suppressors, and the results provide persuasive support for an overall conclusion that "thermodynamic stabilization is neither necessary nor sufficient for suppression." Rather, as summarized in the Discussion, "the findings collectively indicate the role of kinetic stability, in particular an increase in the refolding rate constant as being primarily responsible for the suppressor phenotype."
The authors are seeking an underlying mechanism that explains global suppression, an issue of fundamental importance. Indeed, the finding that enhancement of refolding rates in these suppressors is important, but I don't think it is a "mechanism" in the usual sense of the word. The attendant finding that inactivating mutants largely affect the core while global suppressors localize at the solvent-accessible surface (especially in loops) is both intriguing and suggestive.
What is it about distal loops that can affect global suppression? For what it's worth, an old result from John Gerlt comes to mind. Here, deletion of an entire loop in staphylococcal nuclease resulted in a protein that is significantly more active than wild type. [Poole et al (1991) Deletion of the omega-loop in the active site of staphylococcal nuclease. 1. Effect on catalysis and stability, Biochemistry 30, 3621-3627. Baldisseri et al (1991) Deletion of the omega-loop in the active site of staphylococcal nuclease. 2. Effects on protein structure and dynamics, Biochemistry 30, 3628-3633.]**

*Response:* We thank the reviewer for pointing this out. Indeed, the suppressors are largely identified in distal loops, far from the inactive mutations which are present on the core. There are various possible explanations. Firstly, the core is well packed, and it may not be easy to find mutations that improve upon this in the context of the wild type protein. In the context of a core PIM, destabilizing effects can be alleviated by proximal suppressors which restore packing/H-bonding. However, as we have discussed previously (Sahoo *et al*, 2015), these will be allele specific, not global suppressors. In contrast to the core, there are fewer constraints for surface residues, especially in flexible loops, hence stabilizing mutations are enriched in such sites. In the specific case of CcdB, the mutations result in new native-state interactions that stabilize the loop and/or surrounding loops. We thank the Reviewer for pointing out the SNase loop deletion studies which also demonstrate that flexible loop regions can be destabilizing, entirely consistent with our results. We have included these insights in first para of page 21 of the “Discussion” section.

**A question that arises is the interaction between the CcdB loop that includes suppressor candidate residues 10-12, and cognate structures, such as CcdA. The authors do raise this issue, but I would have welcomed slightly more structural information about the extent to which protein:protein binding might complicate the interpretation of suppression. Along similar lines, the high resolution structures of S12G, V46L and S60E seem to invite detailed assessment of differences in hydrogen-bonding, solvent accessibility, and possible intramolecular interactions between proximal loops, beyond those shown pictorially in fig. 6.**

*Response:* We thank the reviewer for pointing this out. We have now included information on the conformational change of the 8-15 and 39-52 loop regions in the CcdA bound structure in Supplementary Figure S7. The ccd toxin-antitoxin system is involved in plasmid maintenance and bacterial persistence. The CcdB toxin binds and inhibits the function of homodimeric DNA Gyrase, an important, validated drug target. Both fluoroquinolone drugs and the bacterial toxin CcdB cause cell death through DNA gyrase poisoning. The intrinsically disordered C-terminal domain of the cognate antitoxin CcdA, facilitates the rejuvenation of the poisoned Gyrase-CcdB complex by forming a transient ternary complex prior to extracting CcdB from its complex with Gyrase. Most of the identified suppressors are present in the distal loops 8-15 and 39-52 which are directly involved in CcdA binding. In a separate study, we observe that though the individual suppressor mutations do not greatly alter affinity towards CcdA, mutations at all these positions significantly affect the process of rejuvenation (Chattopadhyay *et al*, 2022) The functional importance of these CcdB residues, explains why the global suppressor mutations that we identified are not found in naturally occurring ccdB genes. These points are now mentioned in middle-last para of page 20 in the Discussion section. More detailed assessments of differences in hydrogen-bonding (Supplementary Table S13), solvent accessibility (Supplementary Table S14), and possible intramolecular interactions between proximal loops (Supplementary Figure S10) are shown in the corresponding Supplementary Figure S9, Supplementary Table S13 and Supplementary Table S14.

**To show I read the paper carefully:**

**lines 294, 296: fold, not "folds", e.g., 10-fold, not "10-folds".**

Correction incorporated

**line 488: To, not "Till"**

*Response:* We have now made the necessary changes

**line 502: delete final comma before the period.**

*Response:* We have now made the necessary changes.

**This is an outstanding paper**

*Response:* We thank the reviewer for a careful review of the manuscript and insightful comments and suggestions. Very motivating for all of us to get these words of appreciation from a distinguished colleague!

**Reviewer #3: In this work Varadarajan and coworkers explore biophysical mechanisms by which global suppressors act on several proteins whose knockout mutations they suppress.
The analysis focuses on two key biophysical factors – thermal stability and folding kinetics using several proteins from various sources and therapeutic areas as example. The authors find that while suppressor mutations often increase stability to compensate for destabilization by detrimental mutations this is not always the case and they note a number of exceptions. In terms of kinetics the authors see a more consistent trend whereby suppressor mutations speed up in vitro folding kinetics both in fast and slow phase and they attribute the rescue effect primarily to kinetic rather than thermodynamic stabilization though direct link between kinetic stabilization and functional effect of suppressors in vivo has not been established in this work. However in discussion they speculate that somehow kinetic stabilization might lead to increase in abundance of functional proteins in the cell, perhaps through the effect on cotranslational folding mediated by non-ATP dependent chaperones.**

**The paper is interesting and the analysis is careful and comprehensive and I recommend publication with enthusiasm after the following concerns are considered:**

**1) The authors emphasize that effect on kinetic rather than thermodynamic stability determine suppressor mutations. Figure 8 is supposed to convey this message but its presentation is cursory, even most notations are not defined and it does not impress on the reader that thermodynamic effects of suppressors are less significant than kinetic ones. E.g. amplitudes a_0 and A_0 are not defined and presentation of Fig.8 appears only in the Discussion. I am not sure therefore how Fig.8 conveys main message of the paper.**

*Response:* We thank the reviewer for pointing this out. We have now altered the legend and included descriptions of the annotations. We have also included the numerical values of the mean changes in thermodynamic and kinetic parameters in Table S11. Figure 8 is mentioned in the Discussion rather than the Results because it represents the mean values of changes in the various parameters taken from measurements across different proteins. Supplementary Table S12 and Supplementary Figure S8 depict the average changes in stability and folding kinetic parameters separately for three individual proteins, namely CcdB, TEM-1 β-lactamase, and mRBD respectively. Figure 7 provides additional evidence in support of the important role of folding kinetics as a determinant of suppressor phenotypes

**2) In Fig.1 kinetic traces are provided but no analysis of rates, amplitudes etc are given**

*Response:* Figure 1 does not describe any kinetic data. Instead, changes in thermodynamic stability parameters for the various mutants relative to WT are shown in Figure 1C and in Table S2. Parameters associated with the kinetic traces of Figure 2A-B are listed in Supplementary Table S3 and extrapolated values at zero denaturant in Supplementary Table S4. These points are now mentioned in the corresponding figure captions.

**3) The authors suggest that the effect of mutations on vitro kinetics may indicate a more profound effect on cotranslational folding and resulting yields of functional proteins. Recent paper PMID: 31911473 provides evidence of direct link between kinetic traps manifest in biphasic folding kinetics in vitro and effects on cotranslational folding. In this sense it may be worthwhile to explore the linear association between PIM and/or loci of rescue mutations and slow (for E. coli) codons in endogenous Ec genes such as ccdB.**

*Response:* We thank the reviewer for pointing us to this interesting work. The present study does not directly shed light on mutational effects on co-translational folding or the role of co-translational folding in alleviating kinetic traps. However, since destabilizing mutations at buried sites typically slow down the folding process *in vitro*, we speculate that this also holds true *in vivo*. In the crowded environment of the cell this might facilitate off-pathway aggregation. Suppressor mutations that accelerate refolding can counteract this, enhancing the yield of properly folded, functional protein *in vivo*. We have carried out deep mutational scans of both ccdA (Chandra *et al*, 2022) and ccdB (Bajaj *et al*, 2022) genes in their operonic context. For ccdA which has a large, intrinisically disordered region, we observe phenotypic effects of mutations throughout the protein that are related to codon preference. In case of the present study, the normalised codon usage frequency for the five inactive mutants and the six suppressors for the endogenous *E. coli* CcdB gene are as follows:

| **Position** | **Codon** | **Amino acid** | **Codon usage frequency** | **Normalised codon usage frequency_0 to 1** |
| --- | --- | --- | --- | --- |
| 5 | GTT | V | 0.28 | 0.6 |
| 8 | TAT | Y | 0.59 | 1.0 |
| 11 | GAG | E | 0.32 | 0.0 |
| 12 | AGC | S | 0.25 | 1.0 |
| 18 | GTG | V | 0.35 | 1 |
| 20 | GTA | V | 0.17 | 0 |
| 32 | ATG | M | 1 | 1 |
| 36 | CTG | L | 0.47 | 1 |
| 46 | GTC | V | 0.2 | 0.17 |
| 60 | AGC | S | 0.25 | 1 |
| 83 | CTC | L | 0.1 | 0.14 |

The inactive mutants and the suppressors are highlighted in red and blue colour respectively. The normalised codon usage frequency of 1 indicates that the most preferred codon is present in the original WT CcdB gene sequence and 0 indicates that the least preferred codon is present in the WT CcdB gene sequence. We observe that of the five inactive mutants, at three positions, the original WT codon is the most preferred and at two positions, it is the least preferred codon. For six of the suppressors used in this study, we observe that at four positions, the original WT codon is the most preferred and at the remaining two positions it is the least preferred. We performed a linear regression analysis of the kinetic rate constant parameters with the normalised codon usage frequency for six of the mutants where it was possible to infer the rate constants at zero denaturant concentration. We observe poor correlation between the rate constants of refolding and unfolding with the normalised codon usage frequencies (Figure R1). This could be because codon usage plays little role in the observed *in vivo* folding defects of the inactive mutants or their rescue by the suppressor substitutions. Alternatively, a larger dataset is likely required to address this issue. We have therefore not discussed this issue in the present manuscript.

**Figure R1. Correlation between kinetic rate constants associated with the inactive and suppressor mutations, and the corresponding normalised codon usage frequency at that position.** The rate constants for refolding of the fast phase kf_1_ (red), slow phase kf_2_ (blue) and unfolding ku_1_ (green) are shown. No correlation is observed between these kinetic parameters and the Normalized Codon usage frequency.

**4) The authors point out to examples of stabilizing rescue mutations. However, one the most striking stabilizing mutations that gives rise to evolution of antibiotic (TMP) resistance is L28R in Ec DHFR. A 2016 paper PMID: 26929328 provides a detailed biophysical analysis of this stabilizing rescue mutation and its epistatic effects on abundance, activity, antibiotic binding etc and possible tradeoffs including effects of chaperones and proteases.**

*Response:* We thank the reviewer for pointing this out. We have now included the following statement in first para of page 22 of the “Discussion” section. “Furthermore, the effect of overexpression of GroEL/ES chaperonin system and the deletion of Lon protease on the fitness of a stabilized mutant L28R in the background of WT and two destabilized mutants P21L, A26T was investigated for the trimethoprim (TMP) resistance of *Escherichia coli* dihydrofolate reductase (DHFR). It was observed that the levels of GroEL/ES chaperonins and Lon protease affects the intracellular steady-state concentration of DHFR in a mutation-specific manner and there are complex, epistatic interactions between the three mutations (Rodrigues *et al*, 2016).”

**References**

Bajaj P, Manjunath K & Varadarajan R (2022) Structural and functional determinants inferred from deep mutational scans. *Protein Sci.* **31:** e4357

Chandra S, Manjunath K, Ashok A & Varadarajan R (2022) Inferring bound structure and residue specific contributions to binding energetics in the Intrinsically Disordered Protein, CcdA. *bioRxiv***:** 2022.04.08.487678

Chattopadhyay G, Ahmed S, Srilatha NS, Ashok A & Varadarajan R (2022) Ter-Seq: A high-throughput method to stabilize transient ternary complexes and measure associated kinetics. *bioRxiv***:** 2022.05.18.492574

Rodrigues J V, Bershtein S, Li A, Lozovsky ER, Hartl DL & Shakhnovich EI (2016) Biophysical principles predict fitness landscapes of drug resistance. *Proc. Natl. Acad. Sci. U. S. A.* **113:** E1470-8

Sahoo A, Khare S, Devanarayanan S & Jain PC (2015) Residue proximity information and protein model discrimination using saturation-suppressor mutagenesis. *Elife* **4:** e09532

Sideraki V, Huang W, Palzkill T & Gilbert HF (2001) A secondary drug resistance mutation of TEM-1 β-lactamase that suppresses misfolding and aggregation. *Proc. Natl. Acad. Sci.* **98:** 283–288

Wallentine BD, Wang Y, Tretyachenko-Ladokhina V, Tan M, Senear DF & Luecke H (2013) Structures of oncogenic, suppressor and rescued p53 core-domain variants: Mechanisms of mutant p53 rescue. *Acta Crystallogr. Sect. D Biol. Crystallogr.* **69:** 2146–2156

Wang X, Minasov G & Shoichet BK (2002) Evolution of an antibiotic resistance enzyme constrained by stability and activity trade-offs. *J. Mol. Biol.* **320:** 85–95

Wong KB, DeDecker BS, Freund SM, Proctor MR, Bycroft M & Fersht AR (1999) Hot-spot mutants of p53 core domain evince characteristic local structural changes. *Proc. Natl. Acad. Sci. U. S. A.* **96:** 8438–42
